# Supplementary material for: Screening uptake of colonoscopy versus fecal immunochemical testing in first-degree relatives of patients with non-syndromic colorectal cancer: A multicenter, open-label, parallel-group, randomized trial (ParCoFit study)
Source: PLoS Med. 2023 Oct 24;20(10):e1004298. doi: 10.1371/journal.pmed.1004298 (PMC10597530; doi:10.1371/journal.pmed.1004298)
Supplement: S1 CONSORT Checklist — (DOC) [file pmed.1004298.s001.doc]

CONSORT 2010 checklist of information to include when reporting a randomised trial*

| Section/Topic | Item No | Checklist item | Reported on page No |
| --- | --- | --- | --- |
| Title and abstract | | | |
|  | 1a | Identification as a randomised trial in the title | Title |
|  | 1b | Structured summary of trial design, methods, results, and conclusions (for specific guidance see CONSORT for abstracts) | Abstract |
| Introduction | | | |
| Background and objectives | 2a | Scientific background and explanation of rationale | Introduction, paragraphs 1-4 |
|  | 2b | Specific objectives or hypotheses | Introduction, para 5 |
| Method | | | |
| Trial design | 3a | Description of trial design (such as parallel, factorial) including allocation ratio | Methods, para 2 (selection process) |
|  | 3b | Important changes to methods after trial commencement (such as eligibility criteria), with reasons | Statistical analysis and sample size calculation. para 4  Futility analysis, because low recruitment rate |
| Participants | 4a | Eligibility criteria for participants | Methods, para 1 (Study population) |
|  | 4b | Settings and locations where the data were collected | Methods, para 4 (Study procedures) |
| Interventions | 5 | The interventions for each group with sufficient details to allow replication, including how and when they were actually administered | Methods, para 1 (Selection process and study interventions) |
| Outcomes | 6a | Completely defined pre-specified primary and secondary outcome measures, including how and when they were assessed | Methods, (outcomes) |
|  | 6b | Any changes to trial outcomes after the trial commenced, with reasons | n/a |
| Sample size | 7a | How sample size was determined | Methods, para 4 (Statistical analysis and sample size section) |
|  | 7b | When applicable, explanation of any interim analyses and stopping guidelines | Statistical analysis and sample size calculation. para 4  Futility analysis, because low recruitment rate |
| Randomisation: |  |  |  |
| Sequence generation | 8a | Method used to generate the random allocation sequence | Methods, para 1 (Selection process and study interventions) |
|  | 8b | Type of randomisation; details of any restriction (such as blocking and block size) | Methods, para 1 (Selection process and study interventions) |
| Allocation concealment mechanism | 9 | Mechanism used to implement the random allocation sequence (such as sequentially numbered containers), describing any steps taken to conceal the sequence until interventions were assigned | Methods, para 1 (Selection process and study interventions) |
| Implementation | 10 | Who generated the random allocation sequence, who enrolled participants, and who assigned participants to interventions | Methods, para 1 (Selection process and study interventions) |
| Blinding | 11a | If done, who was blinded after assignment to interventions (for example, participants, care providers, those assessing outcomes) and how | Methods, para 1 (Selection process and study interventions) |
|  | 11b | If relevant, description of the similarity of interventions | n/a |
| Statistical methods | 12a | Statistical methods used to compare groups for primary and secondary outcomes | Methods (statistical analysis) |
|  | 12b | Methods for additional analyses, such as subgroup analyses and adjusted analyses | Methods (statistical analysis) |
| Results | | | |
| Participant flow (a diagram is strongly recommended) | 13a | For each group, the numbers of participants who were randomly assigned, received intended treatment, and were analysed for the primary outcome | Results (para 1), Figure 1 |
|  | 13b | For each group, losses and exclusions after randomisation, together with reasons | Figure 1 |
| Recruitment | 14a | Dates defining the periods of recruitment and follow-up | Results, para 1 (Study population) |
|  | 14b | Why the trial ended or was stopped | Methods, (Statistical analysis), para 4 |
| Baseline data | 15 | A table showing baseline demographic and clinical characteristics for each group | Table 1 |
| Numbers analysed | 16 | For each group, number of participants (denominator) included in each analysis and whether the analysis was by original assigned groups | Tables 1,3-5, 7,8 |
| Outcomes and estimation | 17a | For each primary and secondary outcome, results for each group, and the estimated effect size and its precision (such as 95% confidence interval) | Tables 3, 5, 7 and 8  Outcomes and follow-up |
|  | 17b | For binary outcomes, presentation of both absolute and relative effect sizes is recommended | Tables 3, 5, 7 and 8  Outcomes and follow-up |
| Ancillary analyses | 18 | Results of any other analyses performed, including subgroup analyses and adjusted analyses, distinguishing pre-specified from exploratory | Results, demographic data in non-compliants, Table 2. As screened-analysis, Table 4. Follow-up data, Table 6. As screened analysis Diagnostic yield, Table 9 |
| Harms | 19 | All important harms or unintended effects in each group (for specific guidance see CONSORT for harms) | n/a |
| Discussion | | | |
| Limitations | 20 | Trial limitations, addressing sources of potential bias, imprecision, and, if relevant, multiplicity of analyses | Discussion, para 3 (limitations) |
| Generalisability | 21 | Generalisability (external validity, applicability) of the trial findings | Discussion para 2 (strengths) |
| Interpretation | 22 | Interpretation consistent with results, balancing benefits and harms, and considering other relevant evidence | Discussion  Para 4-9 |
| Other information | | |  |
| Registration | 23 | Registration number and name of trial registry | Abstract and methods (para 1) |
| Protocol | 24 | Where the full trial protocol can be accessed, if available | Provided as supportive information |
| Funding | 25 | Sources of funding and other support (such as supply of drugs), role of funders | Submitted with paper |

*We strongly recommend reading this statement in conjunction with the CONSORT 2010 Explanation and Elaboration for important clarifications on all the items. If relevant, we also recommend reading CONSORT extensions for cluster randomised trials, non-inferiority and equivalence trials, non-pharmacological treatments, herbal interventions, and pragmatic trials. Additional extensions are forthcoming: for those and for up to date references relevant to this checklist, see [www.consort-statement.org](http://www.consort-statement.org/).
