# Supplementary material for: Screening uptake of colonoscopy versus fecal immunochemical testing in first-degree relatives of patients with non-syndromic colorectal cancer: A multicenter, open-label, parallel-group, randomized trial (ParCoFit study)
Source: PLoS Med. 2023 Oct 24;20(10):e1004298. doi: 10.1371/journal.pmed.1004298 (PMC10597530; doi:10.1371/journal.pmed.1004298)
Supplement: S1 Text — (DOCX) [file pmed.1004298.s002.docx]

# This supplement contains the following items:

## Original protocol, final protocol, summary of changes (see pages 1 to 15)

- Original statistical analysis plan, final statistical analysis plan, summary of changes (see pages 10, 14-15)

**STUDY PROTOCOL**

Version January 15, 2016, with amendments of December 2, 2019 and September 15, 2020

**" Uptake to Colorectal Cancer Screening in the Familial-risk Population: comparison of annual fecal immunochemical testing versus one-time**

**colonoscopy” (ParCoFit study)**

### Principal investigator:

Enrique Quintero (Canary Islands)

### Local coordinators:

- Antonio Z. Gimeno-Garcia (Canary Islands)
- Alberto Herreros de Tejada (Madrid)
- Angel Lanas (Aragón)
- Francesc Balaguer (Cataluña)
- Joaquin Cubiella (Galicia)
- Luis Bujanda (Basque Country)
- Rodrigo Jover (Valencia)


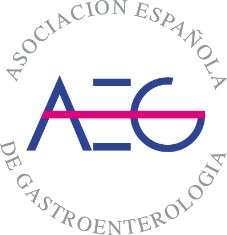


### Index:

|  | Page |
| --- | --- |
| 1. EXECUTIVE SUMMARY | 4 |
| 2. BACKGROUND AND CURRENT STATUS OF THE ISSUE. | 5 |
| 3. OUTCOMES | 7 |
| 4. RESEARCH METHODOLOGY | 7 |
| - Study design | 7 |
| - Target population and study groups | 8 |
| - Selection process and screening invitation. | 8 |
| - Study variables and procedures. | 9 |
| - Statistical analysis and sample size calculation | 10 |
| - Cost and cost-effectiveness analysis. | 10 |
| 5. EXPECTED RESULTS | 11 |
| 6. RESEARCH GROUP EXPERIENCE AND SUITABILITY. | 12 |
| 7. WORKPLAN AND SCHEDULE | 12 |
| 1. COMMUNICATION, DIFFUSION AND APPLICATION OF RESULTS 2. AMENDMENTS | 13  14 |

1. **EXECUTIVE SUMMARY**

**Background:** Colonoscopy is the gold standard procedure for CRC screening in the familial-risk population. However, the efficacy of screening in first-degree relatives (FDR) of patients with colorectal cancer (CRC) is limited by a poor adherence (<50%). Recently it has been suggested that annual fecal immunochemical testing (FIT) is equivalent to colonoscopy for the detection of advanced colorectal neoplasia, if would have the capacity to improve colonoscopy uptake.

**Outcomes:** the primary outcome is to compare screening uptake of annual fecal immunochemical testing (FIT) versus one-time colonoscopy in individuals with a high-risk family history of non- syndromic CRC. Secondary outcomes: 1) to compare the efficacy of annual FIT vs direct colonoscopy for detecting advanced colorectal neoplasia (ACN: advanced adenoma, advanced serrated polyp or CRC) in this population. 2) to determine the cost-effectiveness of both strategies.

**Methodology:** This is a multicentre (12 hospitals), randomized, open-label, parallel group, phase III controlled trial. Inclusion criteria:

1. Having a high-risk family history of CRC: must meet one of the following conditions; a) at least one FDR index case of CRC at <60 years old; b) two FDR diagnosed with CRC, regardless of age at diagnosis; or c) to have a sibling with CRC, regardless of age at diagnosis;
2. Asymptomatic individuals over 40 years old or 10 years younger than the FDR index case when diagnosed with CRC, if the index case was <50 years old.
3. Histologically confirmed CRC in the index case.

Exclusion criteria: prior screening, personal history of ACN, hereditary CRC syndrome, abdominal symptoms needing further investigation or severe comorbidity.

Study groups: 1) annual FIT over three years (cutoff ≥ 10 μg Hb/g faeces to indicate colonoscopy); and

2) straightforward colonoscopy.

The recruitment process will be programmed through the index case, that will be interviewed to obtain their CRC family history. FDR will be contacted to make an appointment in the High-risk CRC Clinic of the participant centres. Randomization (1:1) will be performed before signing the informed consent to avoid selection bias. A researcher will be responsible to provide detailed information about the study and getting the informed consent. In case of willingness to participate in the study, the FDR will be randomized to one of the following arms: A) One-time colonoscopy; B) annual FIT (Automated quantitative Fecal Immunochemical Test) for three screening rounds and a colonoscopy in case of a positive FIT (cut-off = 10 μg Hb/g faeces). Screening uptake will be defined as the percentage of FDR who participate at least in one of the three FIT screening rounds in the FIT group or who undergo colonoscopy in the other group. Screening uptake will be calculated under the assumption of intention to screen analysis. Detection rates of advanced colorectal neoplasia will be assessed by as-screened analysis.

Sample size calculation

Assuming adherence rates of 0.60 vs 0.50 for FIT and colonoscopy, respectively, an alpha risk = 0.05, a beta risk = 0.10, in two-side tails, 538 FDR are required per group. The recruitment process will be programmed through the index case.

### BACKGROUND AND CURRENT STATUS OF THE ISSUE.

Colorectal cancer (CRC) is the most common malignant tumor in Spain with over 30,000 new cases per year and is the second leading cause of cancer mortality in the country with over 14,000 deaths per year [1]. Survival in patients with CRC depends on the disease stage at diagnosis. If detected at an early stage, treatment is curative in nearly 100% of cases, whereas <10% of patients with metastatic CRC survive for more than 5 years [1]. Family history is, with age, the most important risk factor in CRC development. The risk of developing CRC is almost doubled (RR 1.82; 95% CI 1.47, 2.25) in first- degree relatives (FDRs) of patients diagnosed with CRC when over 60 years old. This risk is increased if the index case is less than 60 years old at diagnosis (RR 2.25; 95% CI 1.85, 2.72) or people have two or more FDRs with CRC (4.25; 95% CI 3.01, 6.02) [2]. In addition, it has been recently found that the risk of advanced adenoma is up to three times higher (OR 3.07; 95% CI 1.5, 6.3) among siblings of patients with CRC than among siblings of subjects without CRC [3].

Currently, colonoscopy is the most widely used method for CRC screening in the familial risk population, as it allows both diagnosis and treatment of premalignant lesions (advanced adenomas) by performing polypectomy. Clinical practice guidelines recommend colonoscopy every 5 years, from the age of 40 or ten years less than the youngest relative at CRC diagnosis [4,5]. This strategy greatly reduces the risk of CRC development in FDRs of a patient previously diagnosed with CRC [6]. However, there are no clinical trials comparing the efficacy of colonoscopy screening with other screening strategies.

Despite being the most widely used screening method in the population at familial risk, colonoscopy has numerous drawbacks. Firstly, between 20-26% of adenomas of any size and up to 6-12% of advanced lesions may be missed. Secondly, it is an expensive procedure that requires intravenous sedation and staff training. Thirdly, it is an invasive technique with risks of serious complications, although these are infrequent. Endoscopic polypectomy has a global rate of post-polypectomy bleeding or intestinal perforation of 0.26% and 0.04% of procedures, respectively [7]. However, risk of complications increases significantly if the polyp is located in the cecum or if the polyp is >20 mm in diameter [8]. Furthermore, beneficial effect of colonoscopy to reduce the incidence and mortality rates of CRC is significantly lower in tumors located in the right colon than in those located in the left colon [9]. Finally, its efficacy as a screening test in FDRs of patients with CRC is limited by poor adherence. Population studies conducted in Spain and Australia have found that only 38% and 20% of FDRs, respectively, receive a screening colonoscopy following the recommended timelines given by clinical practice guidelines [10,11].

Several studies have shown that screening by annual or biennial fecal occult blood test (FOBT) reduces CRC incidence and mortality in the average risk population, which consists of asymptomatic persons

≥50 years old with no family history of the disease [12]. In addition, a Spanish multicentre study in this same population showed that immunochemical fecal occult blood test (FIT) was better accepted than colonoscopy and showed a similar CRC detection efficacy [13]. These satisfactory FIT performance results in the average-risk population suggest it could be an alternative to colonoscopy in the familial risk population, especially if the latter manages to significantly increase adherence to screening.

FIT efficacy in the familial risk population has been examined in several pilot studies, showing a sensitivity ranging from 29-80% for detection of advanced adenoma and CRC, with a specificity of 87% and 96%, respectively [14,15]. The only prospective randomized clinical trial comparing FIT and colonoscopy efficacy in FDRs of patients with CRC, conducted by our group, showed that annual FIT is equivalent to colonoscopy for the detection of advanced colorectal neoplasia according to both per- protocol analysis (OR 1.56, 95% CI 0.95, 2.56) and intention-to-treat analysis (OR 1.41, 95% CI 0.88, 2.26) [16]. However, that study was not designed to analyze screening adherence to both tests. In addition, it did not allow the authors to discern whether FIT detection of advanced colorectal neoplasia in the entire familial risk population can be extrapolated to the higher risk subpopulation. Said

subpopulation consists of FDRs of CRC index cases <60 years old and persons with two or more relatives or at least one sibling diagnosed with CRC, regardless of age at diagnosis.

Approximately 70-80% of CRCs develop from a precursor lesion, the adenomatous polyp, following the 'adenoma-carcinoma sequence', such that the removal of these lesions by polypectomy prevents CRC mortality [17]. This represents a crucial advantage for screening as a preventive strategy that can detect and remove the lesion in the asymptomatic phase. However, one of the most significant advances in recent years in gastrointestinal oncology has been the identification of ‘serrated lesions’ as precursors of CRCs through the so-called ‘serrated pathway’ of carcinogenesis, responsible for up to 15-20% of all CRCs [18]. A recent study in Spain found that the overall prevalence of serrated polyps in the intermediate risk population is 20.8%, 6.5% located in the proximal colon and 1.8% greater than 10 mm [19]. These polyps feature a flat morphology, usually located in the right colon and are often covered with mucus, so their detection is difficult and requires a high level of suspicion and adequate colon cleansing [18]. FIT is very reliable for the detection of CRC following the 'adenoma-carcinoma sequence "[13]. However, its diagnostic accuracy for the detection of serrated polyps with increased risk of malignancy, especially sessile serrated polyps, traditional serrated polyps, and hyperplastic polyps larger than 10 mm, is unknown. Furthermore, prevalence of serrated polyps and their role in colorectal carcinogenesis in the population at high familial risk is unknown. The population at high familial risk consists of: FDRs of individuals with an index case when less than 60 years old, persons with two or more relatives with CRC and persons with at least one sibling diagnosed with CRC, regardless of age at diagnosis.

Finally, CRC screening by annual or biennial FIT, sigmoidoscopy every 5 years or colonoscopy every 10 years is cost-effective in the intermediate-risk population [20]. However, there are no data on cost- effectiveness of these methods in the familial risk population from the perspective of healthcare services and society.

Therefore, the hypothesis of this study is that annual screening by FIT can substantially improve adherence to CRC screening in the population at high familial risk with a similar diagnostic efficiency to colonoscopy. With these assumptions, we propose the implementation of a pragmatic study comparing adherence, diagnostic efficacy and cost-effectiveness using annual FIT screening compared to colonoscopy in FDRs at high risk of developing CRC.

### REFERENCES.

1. Morillas JD, Castells A, Oriol I, Pastor A, Pérez-Segura P, Echevarría JM, et al. The Alliance for the Prevention of Colorectal Cancer in Spain. A civil commitment to society. Gastroenterol Hepatol. 2012 Mar;35(3):109-28. PMID: 22365571.

2. Johns LE, Houlston RS. A systematic review and meta-analysis of familial colorectal cancer risk. Am J Gastroenterol. 2001 Oct;96(10):2992-3003. PMID: 11693338.

3. Ng SC, Lau JY, Chan FK, Suen BY, Leung WK, Tse YK, et al. Increased risk of advanced neoplasms among asymptomatic siblings of patients with colorectal cancer. Gastroenterology. 2013 Mar;144(3):544-50. PMID: 23159367.

4. Castells A, Marzo-Castillejo M, Mascort JJ, Amador FJ, Andreu M, Bellas B, et al. Clinical practice guideline. Prevention of colorectal cancer. 2009 update. Gastroenterol Hepatol. 2009 Dec;32(10):717.e1-58. PMID: 20474100.

5. Lieberman DA, Rex DK, Winawer SJ, Giardiello FM, Johnson DA, Levin TR. Guidelines for colonoscopy surveillance after screening and polypectomy: a consensus update by the US Multi-Society Task Force on Colorectal Cancer. Gastroenterology. 2012 Sep;143(3):844-857. PMID: 22763141.

6. Dove-Edwin I, Sasieni P, Adams J, Thomas HJ. Prevention of colorectal cancer by colonoscopic surveillance in individuals with a family history of colorectal cancer: 16 year, prospective, follow-up study. BMJ. 2005 Nov 5;331(7524):1047. PMID: 16243849

7. Gavin DR, Valori RM, Anderson JT, Donnelly MT, Williams JG, Swarbrick ET. The national colonoscopy audit: a nationwide assessment of the quality and safety of colonoscopy in the UK. Gut. 2013 Feb;62(2):242-9. PMID: 22661458.

8. Rutter MD, Nickerson C, Rees CJ, Patnick J, Blanks RG. Risk factors for adverse events related to polypectomy in the English Bowel Cancer Screening Programme. Endoscopy. 2014 Feb;46(2):90-7. PMID: 24477363.

9. Thosani N, Guha S, Singh H. Colonoscopy and colorectal cancer incidence and mortality. Gastroenterol Clin North Am. 2013 Sep;42(3):619-37. PMID: 23931863.

10. Ait Ouakrim D, Lockett T, Boussioutas A, Hopper JL, Jenkins MA. Screening participation for people at increased risk of colorectal cancer due to family history: a systematic review and meta-analysis. Fam Cancer. 2013 Sep;12(3):459-72. PMID: 23700069.

11. Bujanda L, Sarasqueta C, Zubiaurre L, Cosme A, Muñoz C, Sánchez A, et al. EPICOLON Group. Low adherence to colonoscopy in the screening of first-degree relatives of patients with colorectal cancer. Gut. 2007 Dec;56(12):1714-8. PMID: 17400596

12. Hewitson P, Glasziou P, Watson E, Towler B, Irwig L. Cochrane systematic review of colorectal cancer screening using the fecal occult blood test (hemoccult): an update. Am J Gastroenterol. 2008 Jun;103(6):1541-9. PMID: 18479499.

13. Quintero E, Castells A, Bujanda L, Cubiella J, Salas D, Lanas Á, et al. COLONPREV Study Investigators. Colonoscopy versus fecal immunochemical testing in colorectal-cancer screening. N Engl J Med. 2012 Feb 23;366(8):697-706. PMID: 22356323.

14. Castro I, Cubiella J, Rivera C, González-Mao C, Vega P, Soto S, et al. Fecal immunochemical test accuracy in familial risk colorectal cancer screening. Int J Cancer. 2014 Jan 15;134(2):367-75. PMID: 23818169.

15. Gimeno García AZ, Quintero E, Nicolás Pérez D, Hernández M, Jiménez Sosa A. Colorectal cancer screening in first-degree relatives of colorectal cancer: participation, knowledge, and barriers against screening. Eur J Gastroenterol Hepatol. 2011 Nov;23(12):1165-71. PMID: 21989122.

16. Carrillo-Palau M, Gimeno-García A, Alonso Abreu I, Hernández Guerra M, Nicolás Pérez D, Jiménez-Sosa A, et al. Comparación de la colonoscopia y el test inmunológico de sangre oculta en heces en el cribado del cáncer colorrectal familiar. Gastroenterol Hepatol 2013;36 (3):160.

17. Zauber AG, Winawer SJ, O'Brien MJ, Lansdorp-Vogelaar I, van Ballegooijen M, Hankey BF, et al. Colonoscopic polypectomy and long-term prevention of colorectal-cancer deaths. N Engl J Med. 2012 Feb 23;366(8):687-96. PMID: 22356322

18. Leggett B, Whitehall V. Role of the serrated pathway in colorectal cancer pathogenesis. Gastroenterology. 2010 Jun;138(6):2088-100. PMID: 20420948.

19. Álvarez C, Andreu M, Castells A, Quintero E, Bujanda L, Cubiella J, et al. ColonPrev study investigators. Relationship of colonoscopy-detected serrated polyps with synchronous advanced neoplasia in average-risk individuals. Gastrointest Endosc. 2013 Aug;78(2):333-341.e1. PMID: 23623039.

20. Lansdorp-Vogelaar I, Knudsen AB, Brenner H. Cost-effectiveness of colorectal cancer screening. Epidemiol Rev. 2011;33(1):88-100. PMID: 2163309

### 3. OUTCOMES

Primary outcome: to compare screening uptake of annual fecal immunochemical testing (FIT) versus one-time colonoscopy in individuals with a high-risk family history of non-syndromic CRC.

Secondary outcomes: a) to compare the efficacy of annual FIT vs one-time colonoscopy for detecting advanced colorectal neoplasia (ACN: advanced adenoma, advanced serrated polyp or CRC) in this population. b) to determine the cost-effectiveness of both strategies.

### 3. RESEARCH METHODOLOGY.

#### Study Design:

Multicentre, open-label, parallel group, randomized controlled clinical trial.

**Scope**: The study will be conducted in 7 autonomous communities: Aragon, Canary Islands, Catalonia, the Basque Country, Galicia, Madrid and Valencia.

Participating hospitals: Hospital Universitario de Canarias (Tenerife), Hospital Universitario de Donostia, Compleixo Hospitalario de Ourense, Compleixo Hospitalario de Pontevedra, Compleixo Hospitalario de Vigo, Hospital Universitario Puerta de Hierro, Madrid, Hospital Clinic de Barcelona, Hospital Universitario de Alicante, Hospital Universitario La Fe de Valencia, Hospital del Mar de Barcelona, and Hospital Universitario Lozano Blesa de Zaragoza.

### Target population.

#### Inclusion criteria:

Asymptomatic men and women with one or more FDRs (parents, siblings and children) with a history of CRC, who meet the following characteristics:

1. Asymptomatic individuals meeting one of the following conditions: a) having one index case of CRC diagnosed at <60 years old; b) having two FDR diagnosed with CRC, regardless of age at diagnosis; or c) having a sibling with CRC, regardless of age at diagnosis;
2. FDR over 40 years old or 10 years younger than the index case when diagnosed with CRC, if the index case was <50 years old.
3. Histologically confirmed CRC in the index case.

#### Exclusion criteria:

All individuals who meet the following conditions are to be excluded: 1) To have received previous CRC screening; 2) Personal history of inflammatory bowel disease (ulcerative colitis or Crohn's disease), prior colorectal adenoma or CRC; 3) Family history of familial adenomatous polyposis or hereditary nonpolyposis CRC; 4) Symptoms of colonic disease, such as rectal bleeding, recent change in bowel habits or significant weight loss; 5) Those who cannot undergo colonoscopy due to severe coagulopathy or for having undergone colectomy; 6) Those who have severe comorbidity with poor short-term prognosis (neoplastic disease with an average life expectancy of less than 5 years) or chronic disease with performance status greater than or equal to 2 (2: Self-employed but unable to work and is bedridden <50% of the day time, 3: Requires considerable assistance, frequent medical care and is bedridden >50% of the day time, 4: Is severely disabled and bedridden all the time); and 6) Those who decline to sign the informed consent.

### Study groups.

GROUP 1: Screening by annual FIT for three consecutive years and colonoscopy if the FIT result is

≥10 µg of Hb/g of faeces.

GROUP 2: Screening by one-time colonoscopy. In cases where a full colonoscopy cannot be carried out, CT colonography, capsule colonoscopy or barium enema will be offered instead.

### Selection process and screening invitation.

It will be held on a scheduled basis from index cases diagnosed of CRC during the previous 24 months at the Endoscopy Units of participating centres. After at least 3 months from diagnosis and once radiotherapy or oncologic surgery have been performed, the index case will be contacted by phone to arrange an appointment at the High Risk CRC Clinic (HRCC). In this clinic the patient will be informed of the study objectives and informed consent and permission to contact their FDRs will be requested.

If the patient agrees to participate and meets the inclusion criteria, a family tree of the first generation (parents, children or siblings) will be generated to identify all eligible and living FDRs. At this point, an open-label randomization (1:1) for the subject and investigator, to FIT for three consecutive years and work-up colonoscopy if a positive test versus straightforward colonoscopy will be performed, using the randomization module in RedCap Electronnic Data Capture (REDcap). All individuals will receive a letter signed by the medical coordinator who saw the index case in each hospital. This letter will describe the importance of CRC as a health problem in the population at familial risk and will provide information of the next study in FDRs of patients with CRC (cover letter). Later, a second letter will be sent, also signed by the medical coordinator of the study, in which the individual will be invited to participate in the randomized control study (invitation letter). The study aim and the screening option to which the individual has been assigned will also be included in this second letter. Finally, a phone number or email will be provided to request a visit to the HRCC of the corresponding hospital. Individuals who do not respond to the invitation letter after 2 months will be sent a second invitation letter.

Eligible FDRs that attend the HRCC to participate in the study will receive detailed information on the advantages and disadvantages of both tests. Participants with a negative FIT result will be sent an invitation letter to repeat the test. Crossover between groups will not be allowed.

### Study variables and procedures.

***Colonoscopy***: colonoscopies will be performed by experienced endoscopists who have performed > 200 scans and > 50 polypectomies in the previous year. Sedation and colon cleansing will be performed as previously described (Parra et al. World J Gastroenterol 2006). The Boston classification will be used as a scale for bowel cleansing and it will be considered to be adequate when the score is ≥ 2 points in each segment (Lai EJ, et al Gastrointest Endosc 2009). Each colon segment consists of "ascending colon and cecum", "transverse colon" and "descending colon, sigmoid and rectum ', amounting to a minimum of 6 points in the Boston classification (Lai EJ, et al Gastrointest Endosc 2009). Colonoscopy will be considered complete when cleansing is adequate and the cecum is reached. Otherwise the colonoscopy will be considered incomplete and shall be repeated.

***Polyp features:*** Polyp location will be recorded by segmentation. Polyp size and morphology will be recorded using the Paris classification (Gastrointest Endosc 2003:58, Suppl S3-S43). Whether the polyp was removed whole or by fragments will also be recorded. Lesions will be classified as distal or proximal to the splenic flexure. Adenomas ≥ 10 mm in size, with tubulovillous architecture, with high- grade dysplasia or *in situ* adenocarcinoma -pTis- will be considered to be advanced adenomas. Invasive CRC occurs when neoplastic cells cross the *muscularis mucosae*. Advanced neoplasia is the name given to advanced adenomas or invasive CRC. Serrated polyps will be classified according to the World Health Organization (WHO) classification as hyperplastic polyps, sessile serrated adenomas with or without dysplasia and traditional serrated adenomas. In patients with advanced adenoma >20 mm or with advanced adenoma resected in multiple fragments, a surveillance colonoscopy will be performed at 6-12 months after the previous colonoscopy.

#### Immunochemical Fecal Occult Blood Test (FIT):

Participants assigned to the FIT group will receive an annual automated quantitative FIT kit for three consecutive years. The threshold of 10 μg Hb/g faeces (equivalent to 50 ng Hb/mL buffer) will serve as indication for colonoscopy to be performed. Participants will also receive instructions for home use of the kit, and they will be notified that they must deliver it to the laboratory within 7 days. To perform the test it will not be necessary to follow a diet or restrict the use of drugs. For analysis a participant will be considered compliant if at least one FIT is handed in to the laboratory.

#### Other study variables:

Index case: Sex and age at diagnosis of CRC, lesion location and the anatomopathological result will be recorded.

Family tree in first generation (parents, children or siblings): deceased FDRs and age of living FDRs will be identified. FDRs with colorectal neoplasia will be identified.

FDR epidemiological data: age, sex, rural/urban location, substance abuse, history of CRC or adenoma or both, medical history, history of NSAIDs treatment, aspirin, or anticoagulants will be recorded.

Complications: Serious complications that occur during colonoscopy (those that result in the early termination of the procedure): immediate and delayed post-polypectomy hemorrhage and intestinal perforation will be recorded.

All the study variables will be recorded in an online database provided by the Spanish Gastroenterological Association, a member of the RedCap consortium. This application developed by Vanderbilt University guarantees data confidentiality.

### Statistical analysis plan

Screening uptake and detection rate of advanced neoplasia will be assessed by intention-to-screen analysis. First-degree relatives who will not attend the initial appointment and thus do not provide information about exclusion criteria will be considered as eligible and will be included in the analysis. Non-compliers for the assigned strategy will not be allowed to change to the other group. Between- group comparisons of the main outcomes will be calculated by multivariable logistic-regression analysis with adjustment for age, gender, and centre, and results were reported as odds ratios (OR) with 95% confidence intervals.

Comparisons of continuous variables were performed using the Mann–Whitney U-test. Categorical variables with two categories were compared using the χ^2^ test. All analyses were performed using SPSS statistical software version 25.0.

### Sample size calculation.

The study was designed to achieve a 90% power and 95% confidence level for detecting an increase in the proportion of first-degree relatives undergoing CRC screening of 10% (from 50% in the colonoscopy arm to 60% in the FIT arm). According to these assumptions and considering that up to 5% participants in each group would be lost to follow-up, the estimated sample size was 1076 individuals (538 per arm).

### Cost-Effectiveness Analysis

Cost-effectiveness analysis will consist of a simulation with a Markov model to compare two strategies applicable to CRC prevention in a cohort of 5000 FDRs of patients with CRC: 1) Annual FIT and colonoscopy if the test result is positive; and 2) direct colonoscopy from the start of the screening period.

Transition probabilities between the different states the subjects may undergo will be derived from the literature and results arising from this study. Cost estimates of each event where either screening strategy is performed will be based on those published in the Official Gazette of the Canary Islands. An annual discount rate of 3% will be considered.

The following criteria will be established for model development: 1) The target population (FDRs) will be that defined in the inclusion criteria of this study; 2) In FDRs who are assigned annual FIT, the following transition states will be considered: FIT in the first, second, and third years, colonoscopy after positive FIT results, non-advanced neoplasia diagnosis, advanced neoplasia diagnosis, invasive CRC diagnosis and monitoring; 3) In FDRs who are assigned direct colonoscopy, the transition states will be: colonoscopy in the first year, colonoscopy in the second year if advanced neoplasia of >20 mm is detected, non-advanced neoplasia diagnosis, advanced neoplasia diagnosis, invasive CRC diagnosis and monitoring; 4) Since FIT will be conducted annually, the length of each Markov cycle is set to one year, with a half-cycle correction; 5) A time limit of three years from the perspective of the National Health System will be considered, where only direct healthcare costs are included; 6) Estimated health costs will be: FIT kit, automated analysis, colonoscopy, polypectomy, cost per day of hospital stay when required (polypectomy bleeding, perforation, CRC surgery), cost per surgical procedure (laparoscopy or openlaparotomy) and colectomy; 7) It will be assumed that all lesions detected at colonoscopy are resected and colonoscopies are carried out in full.

The described model will be used to develop a cost analysis including sensitivity analysis of all variables in the model to test its robustness and identify situations that could change the decision strategy. Variables to be considered in the sensitivity analysis are as follows: adherence to the program, adherence to the various rounds of FIT screening, rate of complications, sensitivity and specificity of FIT and discount rate. Cost-effectiveness analysis will be carried out, defining effectiveness as the number of patients in whom a significant colonic lesion (advanced neoplasia) was detected. Calculation of the incremental cost-effectiveness ratio (ICER) will be performed using the following formula:

ICER = Cost b - Cost a

*Effectiveness* b - *Effectiveness* a

where ‘*b*’ is the compared option (annual FIT) and ‘*a*’ is the reference option (one time colonoscopy). ICER is defined as the increase in the average cost of obtaining an additional unit of effectiveness if option "*b*" is used instead of option "a".

#### Probabilistic sensitivity analysis.

Model robustness will be verified with a probabilistic sensitivity analysis using Monte Carlo simulation. For this purpose, the probability distribution functions representing the transition probabilities between states of the Markov model will be specified. Subsequently, results of model cost and effectiveness will be recalculated 10,000 times, taking random values of these distributions every time. Decision analysis, sensitivity analysis and Monte Carlo simulation will be performed with TreeAge (TreeAge Software Inc., Williamstown, Mass, USA).

### EXPECTED RESULTS

Approximately 25% of CRCs occur in first-degree relatives of patients with a history of the disease. With colonoscopy screening it is possible to detect and remove precancerous lesions (advanced adenomatous and serrated polyps) and early CRC. Currently, clinical practice guidelines recommend performing direct colonoscopy for the prevention of this neoplasia. However, compliance with this recommendation is very low, with participation rates below 50%, so now a substantial number of individuals at risk of developing the disease are unprotected. This project is innovative in that it includes a scheduled screening method with a non-invasive procedure (FIT). FIT in this study will target people with a family history of CRC, setting the index case at the heart of the screening process. This new

strategy may substantially improve adherence to screening in this population, increasing the rate of early detection of disease in the short term and resulting in a lower incidence and mortality in the long term. This project explores for the first time the efficacy of annual FIT as an alternative to colonoscopy in this high-risk population, which may represent a significant change in current clinical practice of familial CRC screening.

### RESEARCH GROUP EXPERIENCE AND SUITABILITY.

The research proposal described here seeks to continue a line of research begun in 2004 and aims to evaluate new strategies for CRC screening, led by Dr. Enrique Quintero at Hospital Universitario de Canarias. This research group is part of the EPICOLON consortium, an initiative led by participating groups, including more than 25 Spanish medical centres with the aim of characterizing hereditary and familial forms of CRC in Spain. Moreover, since 2008 Dr. Quintero co-directs the ColonPrev project, a multicentre study comparing screening efficacy of FIT and colonoscopy to reduce CRC mortality in the intermediate-risk population. Preliminary results of this project have been published in high impact journals (joint publications in recent years listed below).

The researchers who draft this proposal have collaborated in several projects within the EPICOLON consortium for the past ten years and already have extensive experience in the development of multicentre studies related to this issue, as evidenced by their excellent scientific production.

### Publications from collaborative studies of the research group

- Balaguer F, et al. Clin Gastroenterol Hepatol 2007; 5: 379-387

- Bujanda L, et al. Gut 2007; 56: 1714-1718.

- Balaguer F, et al. Gastroenterology 2008; 134:39-46.
- Jover R, et al. Gastroenterology 2011; 140:1174-1181.
- Quintero E, N Engl J Med 2012; 366:697-706.
- Jover R, et al. Endoscopy 2012; 44:444-451.
- Jover R, et al. Gastrointest Endosc 2013. 77:381-9
- Alvarez C, et al. Gastrointest Endosc 2013;78:333-41 e1.
- Castells A, et J Natl Cancer Inst. 2013 Jun 19;105:878-886.
- Castro I, et al. Int J Cancer 2014;134:367-75.
- Bujanda L, et al. Br J Cancer 2014;110:1334-7.
- Alvarez C et al. Gastrointest Endosc 2013;78:333-41

### WORKPLAN AND SCHEDULE. Development stages (study timeline):

**Year 1 (February 2016 to February 2017)**

- Development of study logistics (circuits, database, listings) (1^st^ quarter)
- Staff training (data-managers) (1^st^ quarter)
- Identification of index cases in Endoscopy Units
- Sending information and invitation letters to FDRs (1^st^ to 4^th^ quarter)
- Start of FDR recruitment for the study (2^nd^ to 4^th^ quarter)
- Start of colonoscopies (2^nd^ to 4^th^ quarter)
- Completion of FDR recruitment (4^th^ quarter)
- Completion of the 1^st^ screening round in the FIT group

### Year 2 (February 2017 to February 2018)

- Completion of the 2^nd^ screening round in the FIT group
- Performance of colonoscopies of randomized patients (1^st^ to 4^th^ quarter), colonoscopies of patients with positive FIT results, repetition of incomplete colonoscopies and repetition of colonoscopies in which high-risk polyps were found or where piecemeal resection of these polyps was performed.

### Year 3 (Februry 2018 to February 2019)

- Completion of the 3^rd^ screening round in the FIT group.
- Performance of colonoscopies of randomized patients (1^st^ to 4^th^ quarter), colonoscopies of patients with positive FIT results, repetition of incomplete colonoscopies and repetition of colonoscopies in which high-risk polyps were found or where piecemeal resection of these polyps was performed.
- Cost-effectiveness analysis (3^rd^ and 4^th^ quarter).

### Follow-up (February 2019 to February 2020)

Participants will be actively followed for at least 12 months after the last event registered in the study, in order to detect screening and colorectal neoplasia beyond the recruitment period. In the FIT group an interval colonoscopy is defined as any colonoscopy performed after a negative FIT result. In the colonoscopy group, interval colonoscopy refers to colonoscopies performed within 36 months after a baseline colonoscopy. Unplanned FIT is defined as any FIT performed because abdominal symptoms in both study groups or when it is performed as a screening tool in the colonoscopy group.

Screening tests, interval colonoscopies, unplanned FIT, post-polypectomy surveillance, interval CRC and deaths, will be identified through cross-linkage of the study database and the regional intranet network that provides access to the electronic medical record at each site. Interval cancer is defined as cancer occurring between 6 and 36 months after a negative screening colonoscopy.

**ParCoFit infrastructure and Allocation of tasks**

**The Scientific Committee (SC) and Scientific Advisory Board** composed by the trial local coordinators (AZG, AHT, AL, LB, JC, RJ and FB) in each Autonomous Community, will have overall responsibility and decision authority for the trial in general, including aspects of management, screening, quality control, endpoint observation and publication activity. Reviews and summarizes reports on quality and adverse events from the secretariat. They will monitor and coordinate the work of the data managers and will be responsible for checking data recorded in the online database developed for this purpose (RedCap). In addition, DNP (Canary Islands University Hospital), will be responsible for cost-effectiveness analysis.

**Coordinating secretariat.** The La Laguna secretariat manages the trial together with the national screening sites and is responsible for data collection from all screening centres. The secretariat sets up and manages the ParCoFit trial database, including tracking of data, collected and stored in REDcap hosted at the Asociación Española de Gastroenterología, a database that guarantees data confidentiality (McNicholl AG, Gisbert JP. Research to the N-Power: The Strengths of Networked Clinical Collaboration in Spain. Am J Gastroenterol. 2017;112:1761-1764).

### COMMUNICATION, DIFFUSION AND APPLICATION OF RESULTS.

Currently there are no prospective randomized clinical trials comparing different screening strategies in the high-risk familial CRC population. Completion of this clinical trial will provide evidence on the efficacy and acceptability of FIT screening in this population at high risk of CRC. This is expected to have a significant scientific impact, like that of a similar recent study on the average risk population

performed by the same research group (Quintero et al. New Engl J Med 2012). Furthermore, results of this study will be disseminated by the Oncology Group of the Spanish Oncology Association, by the EPICOLON Consortium and by the Alliance for the Prevention of CRC, entities to which researchers of this project belong. Finally, results of this study will be presented at the National Network for CRC Screening, which will evaluate the possible implementation of a new strategy for CRC prevention in the familial risk population.

### 10. AMENDMENTS Amendment 1

(Approved by the ParCoFit board on December 2, 2019)

1. Study recruitment and Study Termination

The initial Workplan (page 12) stablished that the third screened round in the FIT group and the corresponding screening colonoscopies in the one-time colonoscopy group should be completed between February 2018 and February 2019. However, the recruitment rate was much lower than expected and by December 2019 only 870 out of 1076 (81%) of the sample size had been recruited with similar screening uptake of the FIT group (439/538; 81.6%) and the colonoscopy group (431/538; 80.1%). Due to the low recruitment rate, the board decided to perform an interim analysis of the primary outcome, showing a similar screening uptake in individuals assigned to one-time colonoscopy (147/431; 34.1%) vs FIT 158/439 (35.9%) (OR 1.12; 95% CI 0.84,1.49, p=0.43).

Therefore, on December 31, 2019, when 81% of the estimated sample size was reac hed, the board decided to terminate the study prematurely after an interim analysis for futility (see page 15).

### Amendment 2

(Approved by the ParCoFit board on September 15, 2020)

As stated at the initial Workplan and Schedule (page 12), one year of follow-up (February 2019 to February 2020) was planned to collect information regarding screening uptake and colorectal neoplasia in first degree relatives beyond the recruitment period. Because of the Covid-19 pandemic the board decided on September 15, 2020 to prolong the follow-up period until December 31, 2021. Therefore, participants were actively followed from the last event registered before December 2019 until December 31, 2021. In the FIT group, an interval colonoscopy was defined as any colonoscopy performed after a negative FIT result. In the colonoscopy group, interval colonoscopy referred to colonoscopies performed within 36 months after a baseline colonoscopy. Unplanned FIT was defined as any FIT performed because of abdominal symptoms in both study groups or when it was performed as a screening tool in the colonoscopy group. Screening tests, interval colonoscopies, unplanned FIT, post-polypectomy surveillance, interval colorectal cancer, and deaths were identified through cross- linkage of the study database and the regional intranet network, which provides access to the electronic medical records at each site. Interval cancer was defined as cancer occurring between 6 and 36 months after a negative colonoscopy screening.

### Amendment 3

(Approved by the ParCoFit board on December 2, 2019)

### Statistical Plan Analysis (page 10)

- 1. **Multiple regression analysis**

To assess screening uptake and advanced colorectal neoplasia multivariable logistic- regression analysis was performed with adjustment for age, gender, and centre. Age was categorized as having more or less than 54 years of age, according to the median age of participants. In addition, centres were categorized as high or low recruiters, if they included

more or less than 80 eligible individuals in the study, respectively. The detection rate of advanced colorectal neoplasia was the number of subjects with true positive results divided by the number of eligible subjects.

### Futility analysis

When 81% of the estimated sample size was reached, an interim analysis was conducted because recruitment was much lower than expected. Based on the screening uptake of 870 randomized first-degree relatives, the futility analysis (Chang M. Classical and Adaptive Clinical Trial Designs: John Wiley & Sons. Hoboken, New Yersey; 2008.70-77) provided a conditional power of 2.95% a predictive power of 0.29% and a futility index of 97.1% (Supplementary Table 1). Therefore, on December 31, 2019, the trial Scientific Committee decided to interrupt the study for futility.
