# Supplementary material for: Screening uptake of colonoscopy versus fecal immunochemical testing in first-degree relatives of patients with non-syndromic colorectal cancer: A multicenter, open-label, parallel-group, randomized trial (ParCoFit study)
Source: PLoS Med. 2023 Oct 24;20(10):e1004298. doi: 10.1371/journal.pmed.1004298 (PMC10597530; doi:10.1371/journal.pmed.1004298)
Supplement: S2 Text — (PDF) [file pmed.1004298.s003.pdf]

- 5 MAR 2015

COMITE DE ÉTICA E INVESTIGACIÓN CLÍNICA - C.E.I.C.
ENVIADO [X] RECIBIDO [ ]
Nº

El estudio de investigación titulado: **"Comparación del test de sangre oculta en heces y la colonoscopia en el cribado del cáncer colorrectal familiar: análisis de la adherencia, eficacia diagnóstica y coste-efectividad"**, con código **2014_45**, del que es Investigador Principal el Dr. **ANTONIO Z. GIMENO GARCIA**, ha sido evaluado por el Comité Ético de Investigación Clínica del Hospital Universitario de Canarias en su sesión del **31 de julio de 2014**, y considera que:

Se cumplen los requisitos necesarios de idoneidad del Protocolo con los objetivos del estudio.

El procedimiento para obtener el consentimiento informado, incluyendo la hoja de información para los sujetos y el consentimiento informado, es adecuado.

La capacidad del Investigador y los medios disponibles son adecuados para llevar a cabo el estudio y no interfiere con el respeto a los postulados éticos.

Por todo ello, el Comité Ético de Investigación Clínica del Hospital Universitario de Canarias **Autoriza*** la realización de este estudio.

La Laguna, a 31 de julio de 2014.

Firmado:

Servicio Canario de la Salud
HOSPITAL UNIVERSITARIO DE CANARIAS
COMITE DE ÉTICA E INVESTIGACIÓN CLÍNICA
C.E.I.C.

Dra. CONSUELO MARIA RODRIGUEZ JIMENEZ
Secretaría del Comité Ético de Investigación Clínica
Hospital Universitario de Canarias

Ofra, s/n. La Cuesta
38320 La Laguna. Santa Cruz de Tenerife.
Tel.: 922 67 80 00 - Fax: 922 65 38 08

Z61259

*NOTA ACLARATORIA AL PROMOTOR:

- La realización de este ensayo clínico en nuestro centro estará condicionada a la aportación de la póliza de seguro de responsabilidad civil a este Comité.

La Laguna, a 31 de julio de 2014.

Firmado:

Servicio Canario de la Salud
HOSPITAL UNIVERSITARIO DE CANARIAS
COMITÉ DE ÉTICA E INVESTIGACIÓN CLÍNICA
C.E.I.C.

Dra. CONSUELO MARIA RODRIGUEZ JIMENEZ
Secretaría del Comité Ético de Investigación Clínica
Hospital Universitario de Canarias
