# Supplementary material for: Screening uptake of colonoscopy versus fecal immunochemical testing in first-degree relatives of patients with non-syndromic colorectal cancer: A multicenter, open-label, parallel-group, randomized trial (ParCoFit study)
Source: PLoS Med. 2023 Oct 24;20(10):e1004298. doi: 10.1371/journal.pmed.1004298 (PMC10597530; doi:10.1371/journal.pmed.1004298)
Supplement: S3 Text — (DOCX) [file pmed.1004298.s004.docx]

# Este suplemento contiene los siguientes elementos:

## Protocolo original, protocolo final, resumen de los cambios (véanse las páginas 1 a 15)

- Plan de análisis estadístico original, plan de análisis estadístico final, resumen de los cambios (véanse las páginas 10 y 14 a 15)

**PROTOCOLO DE ESTUDIO**

Versión 15 de enero de 2016, con modificaciones del 2 de diciembre de 2019 y 15 de septiembre de 2020

**"Captación de las pruebas de detección del cáncer colorrectal en la población de riesgo familiar: comparación de las pruebas inmunoquímicas fecales anuales versus las de una sola vez**

**colonoscopia" (estudio ParCoFit)**

### Investigador principal:

Enrique Quintero (Islas Canarias)

### Coordinadores locales:

- Antonio Z. Gimeno-Garcia (Islas Canarias)
- Alberto Herreros de Tejada (Madrid)
- Angel Lanas (Aragón)
- Francesc Balaguer (Cataluña)
- Joaquin Cubiella (Galicia)
- Luis Bujanda (Basque Country)
- Rodrigo Jover (Valencia)

### Índice:

|  | Página |
| --- | --- |
| 1. RESUMEN | 4 |
| 2. ANTECEDENTES Y SITUACIÓN ACTUAL DE LA CUESTIÓN. | 5 |
| 3. OBJETIVOS | 8 |
| 4. METODOLOGÍA DE INVESTIGACIÓN | 8 |
| - Diseño de estudio | 8 |
| - Población objetivo y grupos de estudio | 8 |
| - Proceso de selección e invitación. | 9 |
| - Variables y procedimientos de estudio. | 9 |
| - Análisis estadístico y cálculo del tamaño de la muestra | 10 |
| - Análisis de coste-efectividad. | 10 |
| 5. RESULTADOS PREVISTOS | 11 |
| 6. EXPERIENCIA E IDONEIDAD DEL GRUPO DE INVESTIGACIÓN. | 12 |
| 7. PLAN DE TRABAJO Y CALENDARIO | 12 |
| 1. COMUNICACIÓN, DIFUSIÓN Y APLICACIÓN DE LOS RESULTADOS 2. ENMIENDAS | 13  14 |

1. **RESUMEN**

**Antecedentes:** La colonoscopia es el procedimiento de referencia para la detección del CCR en la población de riesgo familiar. Sin embargo, la eficacia de los exámenes de detección en familiares de primer grado (FPG) de pacientes con cáncer colorrectal (CCR) está limitada por una mala adherencia (<50%). Recientemente se ha sugerido que la prueba inmunoquímica fecal anual (FIT) es equivalente a la colonoscopia para la detección de neoplasia colorrectal avanzada, si tendría la capacidad de mejorar la absorción de la colonoscopia.

**Resultados:** el resultado primario es comparar la absorción de exámenes de detección de pruebas inmunoquímicas fecales anuales (FIT, por sus siglas en inglés) versus una colonoscopia de una sola vez en individuos con antecedentes familiares de alto riesgo de CCR no sindrómico. Resultados secundarios: 1) para comparar la eficacia de la FIT anual frente a la colonoscopia directa para detectar neoplasia colorrectal avanzada (ACN: adenoma avanzado, pólipo serrado avanzado o CCR) en esta población. 2) determinar la rentabilidad de ambas estrategias.

**Metodología:** Se trata de un ensayo controlado de fase III multicéntrico (12 hospitales), aleatorizado, de etiqueta abierta, de grupo paralelo. Criterios de inclusión:

1. Tener antecedentes familiares de CCR de alto riesgo: debe cumplir una de las siguientes condiciones; a) Al menos un caso de CCR con índice FPG a <60 años; b) dos FPG diagnosticados con CCR, independientemente de la edad en el momento del diagnóstico; o c) tener un hermano con CCR, independientemente de la edad en el momento del diagnóstico;
2. Individuos asintomáticos mayores de 40 años o 10 años menores que el caso del índice FPG cuando se les diagnosticó CCR, si el caso índice era < 50 años.
3. Histológicamente confirmado CCR en el caso índice.

Criterios de exclusión: exámenes de detección previos, antecedentes personales de ACN, síndrome hereditario del CCR, síntomas abdominales que necesitan más investigación o comorbilidad grave.

Grupos de estudio: 1 ) FIT anual durante tres años consecutivos (límite de 10 μg de heces Hb/g para indicar colonoscopia); y 2) colonoscopia directa.

El proceso de reclutamiento se programará a través del caso índice, que será entrevistado para obtener sus antecedentes familiares de CRC. Los FPG serán contactados para una cita en la Clínica de Alto Riesgo de los centros participantes. La aleatorización (1:1) se realizará antes de firmar el consentimiento informado, para evitar sesgos de selección. Un investigador será responsable de proporcionar información detallada sobre el estudio y de obtener el consentimiento informado. En caso de voluntad de participar en el estudio, el FPG será aleatorizado a uno de los siguientes brazos: A) Colonoscopia directa; B) FIT anual (prueba cuantitativa automatizada de inmunoquímica fecal) para tres rondas de cribado y una colonoscopia en caso de FIT positivo (corte = 10 μg Hb/g de heces). La adherencia al cribado se definió como el porcentaje de FPG que participa al menos en una de las tres rondas de exámenes de detección de FIT en el grupo FIT o que se realiza colonoscopia en el otro grupo. La adherencia al cribado se calculará mediante análisis por intención de cribado. La tasa de detección de neoplasia colorrectal avanzada se analizará por cribado recibido.

Cálculo del tamaño de la muestra

Asumiendo tasas de adherencia de 0.60 vs 0.50 para FIT y colonoscopia, respectivamente, se requiere un riesgo alfa = 0.05, un riesgo beta = 0.10, en colas de dos lados, 538 FPG por grupo. El proceso de reclutamiento se llevará a cabo a través del caso índice.

### ANTECEDENTES Y SITUACIÓN ACTUAL DE LA CUESTIÓN.

### El cáncer colorrectal (CCR) es el tumor maligno más común en España con más de 30.000 nuevos casos al año y es la segunda causa de mortalidad por cáncer en el país con más de 14.000 muertes al año [1]. La supervivencia en pacientes con CCR depende del estadio de la enfermedad en el momento del diagnóstico. Si se detecta en una fase temprana, el tratamiento es curativo en casi el 100% de los casos, mientras que < 10% de los pacientes con CCR metastásico sobreviven más de 5 años [1]. Los antecedentes familiares son, con la edad, el factor de riesgo más importante en el desarrollo del CCR. El riesgo de desarrollar CCR es casi el doble (RR 1,82; IC del 95%: 1,47, 2,25) en los familiares de primer grado (CDR) de los pacientes diagnosticados con CCR cuando tienen más de 60 años. Este riesgo aumenta si el caso índice es menor de 60 años en el momento del diagnóstico (RR 2,25; IC 1,85, 2,72) o si las personas tienen dos o más CDR con CCR (4,25; IC del 95%: 3,01, 6,02) [2]. Además, recientemente se ha encontrado que el riesgo de adenoma avanzado es hasta tres veces mayor (OR 3,07; IC del 95%: 1,5, 6,3) entre hermanos de pacientes con CCR que entre hermanos de sujetos sin CCR [3].

### Actualmente, la colonoscopia es el método más utilizado para la detección del CCR en la población de riesgo familiar, ya que permite tanto el diagnóstico como el tratamiento de lesiones premalignas (adenomas avanzados) mediante la realización de polipectomía. Las guías de práctica clínica recomiendan la colonoscopia cada 5 años, a partir de los 40 o 10 años menos que el pariente más joven en el momento del diagnóstico del CCR [4,5]. Esta estrategia reduce considerablemente el riesgo de desarrollo de CCR en los CDR de un paciente previamente diagnosticado con CCR [6]. Sin embargo, no hay ensayos clínicos que comparen la eficacia del cribado con colonoscopia frente a otras estrategias de cribado.

### A pesar de ser el método de detección más utilizado en la población con riesgo familiar, la colonoscopia tiene numerosos inconvenientes. En primer lugar, entre el 20-26% de los adenomas de cualquier tamaño y hasta el 6-12% de las lesiones avanzadas pueden pasar desapercibidas. En segundo lugar, es un procedimiento costoso que requiere sedación intravenosa y capacitación del personal. En tercer lugar, se trata de una técnica invasiva con riesgos de complicaciones graves, aunque son poco frecuentes. La polipectomía endoscópica tiene una tasa global de sangrado o perforación intestinal post-polipectomía del 0,26% y del 0,04% de los procedimientos, respectivamente [7]. Sin embargo, el riesgo de complicaciones aumenta significativamente si el pólipo se encuentra en el ciego o si el pólipo tiene un diámetro > 20 mm [8]. Además, el efecto beneficioso de la colonoscopia para reducir las tasas de incidencia y mortalidad del CCR es significativamente menor en los tumores localizados en el colon derecho que en los localizados en el colon izquierdo [9]. Por último, su eficacia como prueba de cribado en CDR de pacientes con CCR está limitada por una mala adherencia. Estudios poblacionales realizados en España y Australia han encontrado que solo el 38% y el 20% de los FPG, respectivamente, reciben una colonoscopia de cribado siguiendo los plazos recomendados por las guías de práctica clínica [10,11].

### Varios estudios han demostrado que el cribado mediante prueba de sangre oculta en heces anual o bienal reduce la incidencia y la mortalidad del CCR en la población de riesgo promedio, que está formada por personas asintomáticas ≥50 años sin antecedentes familiares de la enfermedad [12]. Además, un estudio multicéntrico español en esta población mostró que la prueba inmunoquímica de sangre oculta en heces fue mejor aceptada que la colonoscopia, con una eficacia de detección de CCR similar [13]. Estos resultados satisfactorios del rendimiento del FIT en la población de riesgo medio sugieren que podría ser una alternativa a la colonoscopia en la población de riesgo familiar, especialmente si logra aumentar significativamente la adherencia al cribado.

### La eficacia del FIT en la población de riesgo familiar se ha analizado en varios estudios piloto, mostrando una sensibilidad que oscila entre el 29% y el 80% para la detección de adenoma avanzado y CCR, con una especificidad del 87% y el 96%, respectivamente [14,15]. El único ensayo clínico prospectivo aleatorizado que comparó la eficacia del FIT y la colonoscopia en FPG de pacientes con CCR, realizado por nuestro grupo, mostró que el FIT anual es equivalente a la colonoscopia para la detección de neoplasia colorrectal avanzada según análisis por protocolo (OR 1,56, 95 % IC 0,95, 2,56) y por intención de cribado (OR 1,41, IC 95% 0,88, 2,26) [16]. Sin embargo, ese estudio no fue diseñado para analizar la adherencia al cribado de ambas pruebas. Además, no permitió a los autores discernir si la detección de neoplasia colorrectal avanzada mediante FIT en la población de riesgo familiar puede extrapolarse a la subpoblación de mayor riesgo. Dicha subpoblación, está formada por FPG de casos índice con CCR <60 años y personas con dos o más familiares o al menos un hermano diagnosticado con CCR, independientemente de la edad en el momento del diagnóstico.

### Aproximadamente el 70-80% de los CCR se desarrollan a partir de una lesión precursora, el pólipo adenomatoso, siguiendo la "secuencia adenoma-carcinoma", de modo que la extirpación de estas lesiones mediante polipectomía previene la mortalidad por CCR [17]. Esto representa una ventaja crucial para el cribado como estrategia preventiva que puede detectar y eliminar la lesión en la fase asintomática. Sin embargo, uno de los avances más significativos de los últimos años en oncología gastrointestinal ha sido la identificación de 'lesiones serradas' como precursoras de los CCR a través de la denominada 'vía serrada' de la carcinogénesis, responsable de hasta el 15-20% de todos los CCR. [18]. Un estudio reciente en España encontró que la prevalencia global de pólipos serrados en la población de riesgo intermedio es del 20,8%, el 6,5% localizados en el colon proximal y el 1,8% mayores de 10 mm [19]. Estos pólipos presentan una morfología plana, normalmente se localizan en el colon derecho y suelen estar cubiertos de moco, por lo que su detección es difícil y requiere un alto nivel de sospecha y una adecuada limpieza del colon [18]. El FIT es muy fiable para la detección de CCR siguiendo la "secuencia adenoma-carcinoma" [13]. Sin embargo, se desconoce su precisión diagnóstica para la detección de pólipos serrados con mayor riesgo de malignidad, especialmente pólipos serrados sésiles, pólipos serrados tradicionales y pólipos hiperplásicos mayores de 10 mm. Además, se desconoce la prevalencia de pólipos serrados y su papel en la carcinogénesis colorrectal en la población con alto riesgo familiar.

### Finalmente, la detección del CCR mediante FIT anual o bienal, sigmoidoscopia cada 5 años o colonoscopia cada 10 años es coste-efectiva en la población de riesgo intermedio [20]. Sin embargo, no existen datos sobre la rentabilidad de estos métodos en la población de riesgo familiar desde la perspectiva de los servicios sanitarios y de la sociedad.

### Por tanto, la hipótesis de este estudio es que el cribado anual mediante FIT puede mejorar sustancialmente la adherencia al cribado del CCR en la población de alto riesgo familiar con una eficacia diagnóstica similar a la colonoscopia. Con estos supuestos, proponemos la implementación de un estudio pragmático para comparar la adherencia, la eficacia diagnóstica y la rentabilidad mediante el cribado con FIT anual frente al cribado con colonoscopia en FPG con alto riesgo de desarrollar CCR.

### REFERENCIAS.

1. Morillas JD, Castells A, Oriol I, Pastor A, Pérez-Segura P, Echevarría JM, et al. The Alliance for the Prevention of Colorectal Cancer in Spain. A civil commitment to society. Gastroenterol Hepatol. 2012 Mar;35(3):109-28. PMID: 22365571.

2. Johns LE, Houlston RS. A systematic review and meta-analysis of familial colorectal cancer risk. Am J Gastroenterol. 2001 Oct;96(10):2992-3003. PMID: 11693338.

3. Ng SC, Lau JY, Chan FK, Suen BY, Leung WK, Tse YK, et al. Increased risk of advanced neoplasms among asymptomatic siblings of patients with colorectal cancer. Gastroenterology. 2013 Mar;144(3):544-50. PMID: 23159367.

4. Castells A, Marzo-Castillejo M, Mascort JJ, Amador FJ, Andreu M, Bellas B, et al. Clinical practice guideline. Prevention of colorectal cancer. 2009 update. Gastroenterol Hepatol. 2009 Dec;32(10):717.e1-58. PMID: 20474100.

5. Lieberman DA, Rex DK, Winawer SJ, Giardiello FM, Johnson DA, Levin TR. Guidelines for colonoscopy surveillance after screening and polypectomy: a consensus update by the US Multi-Society Task Force on Colorectal Cancer. Gastroenterology. 2012 Sep;143(3):844-857. PMID: 22763141.

6. Dove-Edwin I, Sasieni P, Adams J, Thomas HJ. Prevention of colorectal cancer by colonoscopic surveillance in individuals with a family history of colorectal cancer: 16 year, prospective, follow-up study. BMJ. 2005 Nov 5;331(7524):1047. PMID: 16243849

7. Gavin DR, Valori RM, Anderson JT, Donnelly MT, Williams JG, Swarbrick ET. The national colonoscopy audit: a nationwide assessment of the quality and safety of colonoscopy in the UK. Gut. 2013 Feb;62(2):242-9. PMID: 22661458.

8. Rutter MD, Nickerson C, Rees CJ, Patnick J, Blanks RG. Risk factors for adverse events related to polypectomy in the English Bowel Cancer Screening Programme. Endoscopy. 2014 Feb;46(2):90-7. PMID: 24477363.

9. Thosani N, Guha S, Singh H. Colonoscopy and colorectal cancer incidence and mortality. Gastroenterol Clin North Am. 2013 Sep;42(3):619-37. PMID: 23931863.

10. Ait Ouakrim D, Lockett T, Boussioutas A, Hopper JL, Jenkins MA. Screening participation for people at increased risk of colorectal cancer due to family history: a systematic review and meta-analysis. Fam Cancer. 2013 Sep;12(3):459-72. PMID: 23700069.

11. Bujanda L, Sarasqueta C, Zubiaurre L, Cosme A, Muñoz C, Sánchez A, et al. EPICOLON Group. Low adherence to colonoscopy in the screening of first-degree relatives of patients with colorectal cancer. Gut. 2007 Dec;56(12):1714-8. PMID: 17400596

12. Hewitson P, Glasziou P, Watson E, Towler B, Irwig L. Cochrane systematic review of colorectal cancer screening using the fecal occult blood test (hemoccult): an update. Am J Gastroenterol. 2008 Jun;103(6):1541-9. PMID: 18479499.

13. Quintero E, Castells A, Bujanda L, Cubiella J, Salas D, Lanas Á, et al. COLONPREV Study Investigators. Colonoscopy versus fecal immunochemical testing in colorectal-cancer screening. N Engl J Med. 2012 Feb 23;366(8):697-706. PMID: 22356323.

14. Castro I, Cubiella J, Rivera C, González-Mao C, Vega P, Soto S, et al. Fecal immunochemical test accuracy in familial risk colorectal cancer screening. Int J Cancer. 2014 Jan 15;134(2):367-75. PMID: 23818169.

15. Gimeno García AZ, Quintero E, Nicolás Pérez D, Hernández M, Jiménez Sosa A. Colorectal cancer screening in first-degree relatives of colorectal cancer: participation, knowledge, and barriers against screening. Eur J Gastroenterol Hepatol. 2011 Nov;23(12):1165-71. PMID: 21989122.

16. Carrillo-Palau M, Gimeno-García A, Alonso Abreu I, Hernández Guerra M, Nicolás Pérez D, Jiménez-Sosa A, et al. Comparación de la colonoscopia y el test inmunológico de sangre oculta en heces en el cribado del cáncer colorrectal familiar. Gastroenterol Hepatol 2013;36 (3):160.

17. Zauber AG, Winawer SJ, O'Brien MJ, Lansdorp-Vogelaar I, van Ballegooijen M, Hankey BF, et al. Colonoscopic polypectomy and long-term prevention of colorectal-cancer deaths. N Engl J Med. 2012 Feb 23;366(8):687-96. PMID: 22356322

18. Leggett B, Whitehall V. Role of the serrated pathway in colorectal cancer pathogenesis. Gastroenterology. 2010 Jun;138(6):2088-100. PMID: 20420948.

19. Álvarez C, Andreu M, Castells A, Quintero E, Bujanda L, Cubiella J, et al. ColonPrev study investigators. Relationship of colonoscopy-detected serrated polyps with synchronous advanced neoplasia in average-risk individuals. Gastrointest Endosc. 2013 Aug;78(2):333-341.e1. PMID: 23623039.

20. Lansdorp-Vogelaar I, Knudsen AB, Brenner H. Cost-effectiveness of colorectal cancer screening. Epidemiol Rev. 2011;33(1):88-100. PMID: 2163309.

**3. OBJETIVOS**

**Objetivo principal:** comparar la adherencia al cribado mediante una prueba inmunoquímicas fecal (FIT) anuales frente a la colonoscopia en personas con antecedentes familiares de alto riesgo de CCR no sindrómico.

**Objetivos secundarios:** a) comparar la eficacia de la FIT anual frente a la colonoscopia de cribado para detectar neoplasia colorrectal avanzada (ACN: adenoma avanzado, pólipo serrado avanzado o CCR) en esta población. b) determinar el coste-efectividad de ambas estrategias.

**3. METODOLOGÍA.**

**Diseño del estudio:**

Ensayo clínico controlado, aleatorizado, multicéntrico, abierto, de grupos paralelos.

Alcance: El estudio se realizará en 7 comunidades autónomas: Aragón, Canarias, Cataluña, País Vasco, Galicia, Madrid y Valencia.

Hospitales participantes: Hospital Universitario de Canarias (Tenerife), Hospital Universitario de Donostia, Compleixo Hospitalario de Ourense, Compleixo Hospitalario de Pontevedra, Compleixo Hospitalario de Vigo, Hospital Universitario Puerta de Hierro, Madrid, Hospital Clinic de Barcelona, Hospital Universitario de Alicante, Hospital Universitario La Fe de Valencia, Hospital del Mar de Barcelona y Hospital Universitario Lozano Blesa de Zaragoza.

**Población de estudio:**

**Criterios de inclusión:**

Hombres y mujeres asintomáticos con uno o más FPG (padres, hermanos e hijos) con antecedentes de CCR, que cumplan con las siguientes características:

1) Individuos asintomáticos que cumplen una de las siguientes condiciones: a) tener un caso índice de CCR diagnosticado antes de los 60 años; b) tener dos FPG diagnosticados de CCR, independientemente de la edad al momento del diagnóstico; o c) tener un hermano con CCR, independientemente de la edad en el momento del diagnóstico;

2) FPG mayor de 40 años o 10 años menor que el caso índice al momento del diagnóstico de CCR, si el caso índice tenía <50 años.

3) CCR histológicamente confirmado en el caso índice.

### Criterio de exclusión:

### Se excluirán todas las personas que cumplan con las siguientes condiciones: 1) Haberse realizado una prueba de cribado para CCR previamente; 2) Antecedentes personales de enfermedad inflamatoria intestinal (colitis ulcerosa o enfermedad de Crohn), adenoma colorrectal previo o CCR; 3) Antecedentes familiares de poliposis adenomatosa familiar o CCR hereditario sin poliposis; 4) Síntomas de enfermedad del colon, como sangrado rectal, cambio reciente en los hábitos intestinales o pérdida de peso significativa; 5) Quienes no puedan someterse a una colonoscopia por coagulopatía severa o por haber sido sometidos a colectomía; 6) Quienes presentan comorbilidad severa con mal pronóstico a corto plazo (enfermedad neoplásica con esperanza de vida promedio menor a 5 años) o enfermedad crónica con estado funcional mayor o igual a 2 (2: Trabajadores por cuenta propia pero sin capacidad para trabajar y estar postrado en cama <50% del tiempo del día, 3: requiere asistencia considerable, atención médica frecuente y estar postrado en cama más del 50% del día, 4: está gravemente discapacitado y postrado en cama todo el tiempo); y 6) Quienes se nieguen a firmar el consentimiento informado.

### Grupos de estudio.

### GRUPO 1: Cribado mediante FIT anual durante tres años consecutivos y colonoscopia si el resultado del FIT es ≥10 µg de Hb/g de heces.

### GRUPO 2: Cribado mediante colonoscopia única. En los casos en los que no se pueda realizar una colonoscopia completa, se ofrecerá en su lugar una colonografía por tomografía computarizada, una colonoscopia con cápsula o un enema de bario.

### Proceso de selección e invitación al cribado.

### Se realizará de forma programada a partir de casos índice diagnosticados de CCR durante los 24 meses anteriores en las Unidades de Endoscopia de los centros participantes. Transcurridos al menos 3 meses desde el diagnóstico y una vez realizada la radioterapia o cirugía oncológica, se contactará telefónicamente con el caso índice para concertar una cita en la Clínica de CCR de Alto Riesgo (CCRH). En esta clínica se informará al paciente de los objetivos del estudio y se solicitará el consentimiento informado y el permiso para contactar a sus FPG.

### Si el paciente acepta participar y cumple con los criterios de inclusión, se generará un árbol genealógico de la primera generación (padres, hijos o hermanos) para identificar a todos los FPG elegibles y vivos. En este punto, se realizará una aleatorización de etiqueta abierta (1:1) para el sujeto y el investigador, para FIT durante tres años consecutivos y colonoscopia si el test es positivo, versus colonoscopia simple, utilizando el módulo de aleatorización en RedCap Electronic Data (REDcap). Todos los FPG recibirán una carta firmada por el coordinador médico que atendió el caso índice en cada hospital. Esta carta describirá la importancia del CCR como problema de salud en la población de riesgo familiar y proporcionará información sobre el estudio (carta de presentación). Posteriormente se enviará una segunda carta, también firmada por el coordinador médico del estudio, en la que se invitará al individuo a participar en el estudio (carta de invitación). En esta segunda carta también se incluirá el objetivo del estudio y la opción de cribado a la que ha sido asignado el individuo. Finalmente se facilitará un número de teléfono o correo electrónico para solicitar la visita al CCRH del hospital correspondiente. Las personas que no respondan a la carta de invitación después de 2 meses recibirán una segunda carta de recordatorio.

### Los FPG elegibles que asistan al HRCC para participar en el estudio recibirán información detallada sobre las ventajas y desventajas de ambas pruebas. A los participantes con un resultado FIT negativo se les enviará una carta de invitación para repetir la prueba. No se permitirá el cruce entre grupos.

### Variables y procedimientos del estudio.

### Colonoscopia: las colonoscopias serán realizadas por endoscopistas experimentados que hayan realizado > 200 exploraciones y > 50 polipectomías en el año anterior. La sedación y la limpieza del colon se realizarán como se describió anteriormente (Parra et al. World J Gastroenterol 2006). Se utilizará la clasificación de Boston como escala para la limpieza intestinal y se considerará adecuada cuando la puntuación sea ≥ 2 puntos en cada segmento (Lai EJ, et al Gastrointest Endosc 2009). Cada segmento de colon consta de "colon ascendente y ciego", "colon transverso" y "colon descendente, sigmoide y recto", lo que supone un mínimo de 6 puntos en la clasificación de Boston (Lai EJ, et al Gastrointest Endosc 2009). Se considerará completa cuando la limpieza sea adecuada y se alcance el ciego, de lo contrario la colonoscopia se considerará incompleta y se repetirá.

### Características de los pólipos: Los pólipos se ubicarán por segmentos. El tamaño y la morfología de los pólipos se registrarán utilizando la clasificación de París (Gastrointest Endosc 2003:58, Suppl S3-S43). También se registrará si el pólipo se extirpó completo o en fragmentos. Las lesiones se clasificarán como distales o proximales al ángulo esplénico. Se considerarán adenomas avanzados los adenomas de tamaño ≥ 10 mm, con arquitectura tubulovellosa, con displasia de alto grado o adenocarcinoma in situ -pTis-. El CCR invasivo ocurre cuando las células neoplásicas sobrepasan la muscular de la mucosa. El término neoplasia avanzada incluye los adenomas avanzados y/o CCR invasivo. Los pólipos serrados se clasificarán según la clasificación de la Organización Mundial de la Salud (OMS) en pólipos hiperplásicos, adenomas serrados sésiles con o sin displasia y adenomas serrados tradicionales. En pacientes con adenoma avanzado >20 mm o con adenoma avanzado resecado en múltiples fragmentos, se realizará una colonoscopia de vigilancia a los 6-12 meses después de la colonoscopia previa.

### Prueba de sangre oculta en heces inmunológica (FIT):

### Los participantes asignados al grupo FIT recibirán un kit cuantitativo automatizado anual durante tres años consecutivos. El umbral para indicar la realización de una colonoscopia será de 10 μg de Hb/g de heces (equivalente a 50 ng de Hb/ml de tampón). Los participantes recibirán instrucciones para el uso doméstico del kit y se les notificará que deben entregarlo al laboratorio dentro de los 14 días. Para realizar la prueba no será necesario seguir una dieta ni restringir el uso de medicamentos. Para el análisis, se considerará que un participante cumple si se entrega al menos un FIT al laboratorio.

### Otras variables de estudio:

### Caso índice: Se registrará sexo y edad al diagnóstico de CCR, localización de la lesión y resultado anatomopatológico.

### Árbol genealógico en primera generación (padres, hijos o hermanos): se identificarán los FPG fallecidos y la edad de vida de los FPG. Se identificarán los FPG con neoplasia colorrectal.

### Datos epidemiológicos de FPG: edad, sexo, ubicación rural/urbana, consumo de tabaco y alcohol, antecedentes de CCR o adenoma o ambos, antecedentes patológicos, antecedentes de tratamiento con AINE, aspirina o anticoagulantes.

### Complicaciones: Se registrarán las complicaciones graves que ocurren durante la colonoscopia (aquellas que resultan en la terminación temprana del procedimiento): hemorragia postpolipectomía inmediata o tardía, y perforación intestinal.

### Todas las variables del estudio quedarán registradas en una base de datos online proporcionada por la Asociación Española de Gastroenterología, miembro del consorcio RedCap. Esta aplicación desarrollada por la Universidad de Vanderbilt garantiza la confidencialidad de los datos.

### Análisis estadístico

### La adherencia al cribado y la tasa de detección de neoplasia avanzada se evaluarán mediante un análisis de intención de cribado. Los FPG que no asistan a la cita inicial y por tanto no proporcionen información sobre los criterios de exclusión serán considerados elegibles y serán incluidos en el análisis. Los que no cumplan con la estrategia asignada no podrán cambiar de grupo. Las comparaciones entre grupos de los resultados principales se calcularán mediante análisis de regresión logística multivariable con ajuste por edad, sexo y centro, y los resultados se describirán como odds ratios (OR) con intervalos de confianza del 95%.

### Las comparaciones de variables continuas se realizaron mediante la prueba U de Mann-Whitney. Se compararán variables categóricas con dos categorías mediante la prueba de χ2. Todos los análisis se realizarán utilizando el software estadístico SPSS versión 25.0.

### Cálculo del tamaño muestral.

### El estudio fue diseñado para lograr una potencia del 90% y un nivel de confianza del 95% para detectar un aumento de la adherencia al cribado del 10% en familiares de primer grado invitados a FIT (50% en el grupo de colonoscopia y 60% en el grupo FIT). De acuerdo con estos supuestos y considerando que hasta el 5% de los participantes de cada grupo se perderían durante el seguimiento, el tamaño de muestra estimado fue de 1076 individuos (538 por brazo).

### Análisis de coste-efectividad

### El análisis de coste-efectividad consistirá en una simulación con un modelo de Markov para comparar dos estrategias aplicables a la prevención del CCR en una cohorte de 5000 FPG de pacientes con CCR: 1) FIT anual y colonoscopia si el resultado de la prueba es positivo; y 2) colonoscopia directa desde el inicio del período de selección.

### Las probabilidades de transición entre los diferentes estados que pueden experimentar los sujetos se derivarán de la literatura y de los resultados que surjan de este estudio. Las estimaciones de costes de cada evento en el que se realice cualquiera de las estrategias de cribado se basarán en los publicados en el Boletín Oficial de Canarias. Se considerará una tasa de descuento anual del 3%.

### Se establecerán los siguientes criterios para el desarrollo del modelo: 1) La población objetivo (FPG) será la definida en los criterios de inclusión de este estudio; 2) En los FPG a los que se les asigne FIT anual, se considerarán los siguientes estados de transición: FIT en el primer, segundo y tercer año, colonoscopia después de resultados positivos de FIT, diagnóstico de neoplasia no avanzada, diagnóstico de neoplasia avanzada, diagnóstico y seguimiento de CCR invasivo; 3) En los FPG a quienes se les asigne colonoscopia directa, los estados de transición serán: colonoscopia en el primer año, colonoscopia en el segundo año si se detecta neoplasia avanzada >20 mm, diagnóstico de neoplasia no avanzada, diagnóstico de neoplasia avanzada, diagnóstico de CCR invasivo. y monitoreando; 4) Dado que el FIT se realizará anualmente, la duración de cada ciclo de Markov se fija en un año, con una corrección de medio ciclo; 5) Se considerará un límite temporal de tres años desde la perspectiva del Sistema Nacional de Salud, donde sólo se incluyen los costes sanitarios directos; 6) Los costos de salud estimados serán: kit del FIT, análisis automatizado, colonoscopia, polipectomía, costo por día de estadía hospitalaria cuando se requiera (sangrado por polipectomía, perforación, cirugía de CCR), costo por procedimiento quirúrgico (laparoscopia o laparotomía abierta) y colectomía; 7) Se asumirá que todas las lesiones detectadas en la colonoscopia se resecan y las colonoscopias se realizan en su totalidad.

### El modelo descrito se utilizará para desarrollar un análisis de costes que incluya un análisis de sensibilidad de todas las variables del modelo para probar su solidez e identificar situaciones que podrían cambiar la estrategia de decisión. Las variables a considerar en el análisis de sensibilidad son las siguientes: adherencia al programa, adherencia a las distintas rondas de cribado FIT, tasa de complicaciones, sensibilidad y especificidad del FIT y tasa de descuento. Se realizará un análisis coste-efectividad, definiendo efectividad como el número de pacientes en los que se detectó una lesión colónica significativa (neoplasia avanzada). El cálculo del ratio coste-efectividad incremental (ICER) se realizará mediante la siguiente fórmula:

ICER = Cost b - Cost a

*Effectiveness* b - *Effectiveness* a

### donde 'b' es la opción comparada (FIT anual) y 'a' es la opción de referencia (colonoscopia única). ICER se define como el incremento en el costo promedio de obtener una unidad adicional de efectividad si se utiliza la opción "b" en lugar de la opción "a".

### Análisis probabilístico de sensibilidad.

### La robustez del modelo se verificará con un análisis probabilístico de sensibilidad mediante simulación de Monte Carlo. Para ello se especificarán las funciones de distribución de probabilidad que representan las probabilidades de transición entre estados del modelo de Markov. Posteriormente, los resultados del coste y la eficacia del modelo se recalcularán 10.000 veces, tomando cada vez valores aleatorios de estas distribuciones. Se realizarán análisis de decisión, análisis de sensibilidad y simulación de Monte Carlo con TreeAge (TreeAge Software Inc., Williamstown, Mass, EE. UU.).

### 5. RESULTADOS ESPERADOS

### Aproximadamente el 25% de los CCR ocurren en familiares de primer grado de pacientes con antecedentes de la enfermedad. Con el cribado mediante colonoscopia es posible detectar y extirpar lesiones precancerosas (pólipos adenomatosos y serrados avanzados) y CCR precoz. Actualmente, las guías de práctica clínica recomiendan la realización de colonoscopia directa para la prevención de esta neoplasia. Sin embargo, el cumplimiento de esta recomendación es muy bajo, con tasas de participación inferiores al 50%, por lo que ahora un número sustancial de personas en riesgo de desarrollar la enfermedad se encuentran desprotegidas. Este proyecto es innovador porque incluye un método de detección programado con un procedimiento no invasivo (FIT). El FIT en este estudio se dirigirá a personas con antecedentes familiares de CCR, ubicando el caso índice en el centro del proceso de detección. esta nueva estrategia puede mejorar sustancialmente la adherencia al cribado en esta población, aumentando la tasa de detección precoz de la enfermedad a corto plazo y dando como resultado una menor incidencia y mortalidad a largo plazo. Este proyecto explora por primera vez la eficacia de la FIT anual como alternativa a la colonoscopia en esta población de alto riesgo, lo que puede representar un cambio significativo en la práctica clínica actual de detección del CCR familiar.

### 6. EXPERIENCIA E IDONEIDAD DEL GRUPO DE INVESTIGACIÓN.

### La propuesta de investigación aquí descrita es la continuación de una línea de investigación iniciada en 2004, liderada por el Dr. Enrique Quintero en el Hospital Universitario de Canarias, y pretende evaluar nuevas estrategias para el cribado del CCR. Este grupo de investigación forma parte del consorcio EPICOLON, una iniciativa liderada por los grupos participantes, entre los que se encuentran más de 25 centros médicos españoles, cuyo principal objetivo es caracterizar las formas hereditarias y familiares de CCR en España. Además, desde 2008 el Dr. Quintero codirige el proyecto ColonPrev, un estudio multicéntrico que compara la eficacia del screening de FIT y colonoscopia para reducir la mortalidad por CCR en la población de riesgo intermedio. Los resultados preliminares de este proyecto se han publicado en revistas de alto impacto (las publicaciones conjuntas de los últimos años se enumeran a continuación).

### Los investigadores que redactan esta propuesta han colaborado en varios proyectos dentro del consorcio EPICOLON durante los últimos diez años y cuentan ya con una amplia experiencia en el desarrollo de estudios multicéntricos relacionados con este tema, como demuestra su excelente producción científica.

### Publications from collaborative studies of the research group

- Balaguer F, et al. Clin Gastroenterol Hepatol 2007; 5: 379-387

- Bujanda L, et al. Gut 2007; 56: 1714-1718.

- Balaguer F, et al. Gastroenterology 2008; 134:39-46.
- Jover R, et al. Gastroenterology 2011; 140:1174-1181.
- Quintero E, N Engl J Med 2012; 366:697-706.
- Jover R, et al. Endoscopy 2012; 44:444-451.
- Jover R, et al. Gastrointest Endosc 2013. 77:381-9
- Alvarez C, et al. Gastrointest Endosc 2013;78:333-41 e1.
- Castells A, et J Natl Cancer Inst. 2013 Jun 19;105:878-886.
- Castro I, et al. Int J Cancer 2014;134:367-75.
- Bujanda L, et al. Br J Cancer 2014;110:1334-7.
- Alvarez C et al. Gastrointest Endosc 2013;78:333-41

### 7. PLAN DE TRABAJO Y CALENDARIO.

### Etapas de desarrollo (cronograma del estudio):

### Año 1 (febrero de 2016 a febrero de 2017)

### - Desarrollo de la logística del estudio (circuitos, base de datos, listados) (1er trimestre)

### - Formación del personal (data-managers) (1er trimestre)

### - Identificación de casos índice en Unidades de Endoscopia

### - Envío de cartas de información y invitación a FPG (1º a 4º trimestre)

### - Inicio del reclutamiento de FPG para el estudio (segundo al cuarto trimestre)

### - Inicio de colonoscopias (2º a 4º trimestre)

### - Finalización del reclutamiento de FPG (cuarto trimestre)

### - Finalización de la 1.ª ronda de selección en el grupo FIT.

### Año 2 (febrero 2017 a febrero 2018)

### - Finalización de la 2ª ronda de selección en el grupo FIT.

### - Realización de colonoscopias de pacientes aleatorizados (1º a 4º trimestre), colonoscopias de pacientes con resultados FIT positivos, repetición de colonoscopias incompletas y repetición de colonoscopias en las que se encontraron pólipos de alto riesgo o en las que se realizó resección fragmentada de estos pólipos.

### Año 3 (febrero 2018 a febrero 2019)

### - Finalización de la 3ª ronda de cribado en el grupo FIT.

### - Realización de colonoscopias de pacientes aleatorizados (1º a 4º trimestre), colonoscopias de pacientes con resultados FIT positivos, repetición de colonoscopias incompletas y repetición de colonoscopias en las que se encontraron pólipos de alto riesgo o en las que se realizó resección fragmentada de estos pólipos.

### - Análisis coste-efectividad (3º y 4º trimestre).

### Seguimiento (febrero 2019 a febrero 2020)

### Los participantes serán seguidos activamente durante al menos 12 meses después del último evento registrado en el estudio, para detectar detección y neoplasia colorrectal más allá del período de reclutamiento. En el grupo FIT, una colonoscopia de intervalo se define como cualquier colonoscopia realizada después de un resultado FIT negativo. En el grupo de colonoscopia, la colonoscopia de intervalo se refiere a las colonoscopias realizadas dentro de los 36 meses posteriores a una colonoscopia inicial. El FIT no planificada se define como cualquier FIT realizado debido a síntomas abdominales en ambos grupos de estudio o cuando se realiza como herramienta de detección en el grupo de colonoscopia.

### Las pruebas de detección, las colonoscopias de intervalo, las pruebas no planificadas, la vigilancia postpolipectomía, el CCR de intervalo y las muertes se identificarán mediante el enlace cruzado de la base de datos del estudio y la red de intranet regional que proporciona acceso a la historia clínica electrónica en cada centro participante. El cáncer de intervalo se define como el cáncer que ocurre entre 6 y 36 meses después de una colonoscopia de detección negativa.

### Infraestructura y Asignación de Tareas

### El Comité Científico (CS) y el Consejo Científico Asesor compuesto por los coordinadores locales del ensayo (AZG, AHT, AL, LB, JC, RJ y FB) en cada Comunidad Autónoma, tendrán la responsabilidad general y la autoridad de decisión para el estudio, incluyendo aspectos de gestión, selección, control de calidad, observación de parámetros y actividad de publicación. Asimismo, seguirán y coordinarán el trabajo de los responsables de los datos y serán responsables de comprobar los datos registrados en la base de datos en línea desarrollada al efecto (RedCap). Además, el DNP (Hospital Universitario de Canarias), será el responsable del análisis de coste-efectividad.

### 8. COMUNICACIÓN, DIFUSIÓN Y APLICACIÓN DE RESULTADOS.

### Actualmente no existen ensayos clínicos prospectivos aleatorizados que comparen diferentes estrategias de cribado en la población de CCR familiar de alto riesgo. La finalización de este ensayo clínico proporcionará evidencia sobre la eficacia y aceptabilidad de la detección FIT en esta población con alto riesgo de CCR. Se espera que esto tenga un impacto científico significativo, como el de un estudio reciente similar sobre la población de riesgo promedio realizado por el mismo grupo de investigación (Quintero et al. New Engl J Med 2012). Además, los resultados de este estudio serán difundidos por el Grupo de Oncología de la Asociación Española de Oncología, por el Consorcio EPICOLON y por la Alianza para la Prevención del CCR, entidades a las que pertenecen los investigadores de este proyecto. Finalmente, los resultados de este estudio serán presentados en la Red Nacional de Detección del CCR, donde se evaluará la posible implementación de una nueva estrategia para la prevención del CCR en la población de riesgo familiar.

### ENMIENDAS

### Enmienda 1

### (Aprobado por el Comité Científico del ParCoFit el 2 de diciembre de 2019)

### 1. Reclutamiento del estudio y finalización del estudio

### El Plan de Trabajo inicial (página 12) establecía que la tercera ronda de cribado en el grupo FIT y las correspondientes colonoscopias de cribado en el grupo de colonoscopia única debían completarse entre febrero de 2018 y febrero de 2019. Sin embargo, la tasa de reclutamiento fue más baja de lo esperado y en diciembre de 2019, solo 870 de 1076 (81%) del tamaño de la muestra habían sido reclutados con un reclutamiento similar en el grupo FIT (439/538; 81,6%) y el grupo de colonoscopia (431/538; 80,1%). Debido a la baja tasa de reclutamiento, el Comité decidió realizar un análisis provisional del resultado primario, que mostró una adherencia similar en individuos asignados a una colonoscopia única (147/431; 34,1%) frente a FIT 158/439 (35,9%) (OR 1,12; IC 95% 0,84;1,49, p=0,43). Por ello, el 31 de diciembre de 2019, cuando se alcanzó el 81% del tamaño de muestra estimado, el Comité decidió finalizar el estudio prematuramente después de un análisis intermedio de futilidad (ver página 15).

### Enmienda 2

### (Aprobado por el Comité Científico del ParCoFit el 15 de septiembre de 2020)

### Como se indicó en el Plan de Trabajo y cronograma inicial (página 12), se planificó un año de seguimiento (de febrero de 2019 a febrero de 2020) para recopilar información sobre la realización de pruebas de detección y la neoplasia colorrectal en familiares de primer grado más allá del período de reclutamiento. Debido a la pandemia de Covid-19, el Comité decidió el 15 de septiembre de 2020 prolongar el período de seguimiento hasta el 31 de diciembre de 2021. Por lo tanto, se siguió activamente a los participantes desde el último evento registrado antes de diciembre de 2019 hasta el 31 de diciembre de 2021. En el grupo FIT, una colonoscopia de intervalo se definió como cualquier colonoscopia realizada después de un resultado FIT negativo. En el grupo de colonoscopia, la colonoscopia de intervalo se refería a las colonoscopias realizadas dentro de los 36 meses posteriores a una colonoscopia inicial. El FIT no planificado se definió como cualquier FIT realizado debido a síntomas abdominales en ambos grupos de estudio o cuando se realizó como prueba de cribado en el grupo de colonoscopia. Las pruebas de cribado, las colonoscopias de intervalo, el FIT no planificado, la vigilancia pospolipectomía, el cáncer colorrectal de intervalo y las muertes se identificaron mediante consulta cruzada entre la base de datos del estudio y la red de intranet regional, que brinda acceso a los registros médicos electrónicos en cada centro. El cáncer de intervalo se definió como el cáncer que ocurre entre 6 y 36 meses después de una colonoscopia negativa.

### Enmienda 3

### (Aprobado por el Comité Científico del ParCoFit el 2 de diciembre de 2019)

### Análisis del plan estadístico (página 10)

### a. Análisis de regresión múltiple

### Para evaluar la adherencia al cribado y la neoplasia colorrectal avanzada, se realizó un análisis de regresión logística multivariable con ajuste por edad, sexo, mismas vs diferentes estrategias asignadas por familia, localización del tumor en el caso índice, persona que acudió a la primera cita y centro. Además, los centros se clasificaron como de alto o bajo reclutamiento, si incluían más o menos de 80 personas elegibles en el estudio, respectivamente. La tasa de detección de neoplasia colorrectal avanzada fue el número de sujetos con resultados positivos verdaderos dividido por el número de sujetos elegibles.

### b. Análisis de futilidad

### Cuando se alcanzó el 81% del tamaño de muestra estimado, se realizó un análisis interino debido a que el reclutamiento fue muy inferior al esperado. Basado en la aceptación del cribado de 870 familiares de primer grado aleatorizados, el análisis de futilidad (Chang M. Classical and Adaptive Clinical Trial Designs: John Wiley & Sons. Hoboken, New Yersey; 2008.70-77) mostró una potencia condicional de 2,95 % como valor predictivo. potencia del 0,29% y un índice de futilidad del 97,1% (Tabla complementaria 1). Por ello, el 31 de diciembre de 2019, el Comité Científico del ensayo decidió interrumpir el estudio por inutilidad.
