# Supplementary material for: Screening uptake of colonoscopy versus fecal immunochemical testing in first-degree relatives of patients with non-syndromic colorectal cancer: A multicenter, open-label, parallel-group, randomized trial (ParCoFit study)
Source: PLoS Med. 2023 Oct 24;20(10):e1004298. doi: 10.1371/journal.pmed.1004298 (PMC10597530; doi:10.1371/journal.pmed.1004298)
Supplement: S1 Table — (DOCX) [file pmed.1004298.s005.docx]

**S1 Table. Computation of futility analysis based upon 81% first-degree relatives recruited.**

| Target sample size | Current sample size^a^ | Fixed proportion | Precision | Test Statistic Z_k_^b^ | Conditional Power | Predictive Power | Futility index^c^ |
| --- | --- | --- | --- | --- | --- | --- | --- |
| 538 \| 538 | 439 \| 431 | 0.5 | 0.1 | 0.558 | 2.95% | 0.29% | 97.05% |

^a^ Current sample size: Fecal immunochemical test group (n=439), Colonoscopy group (n=431),

^b^ Z_k_ is the z-statistic computed from the observed data.

^c^A value over 90% indicates that the study should be stopped.
