# Supplementary material for: Screening uptake of colonoscopy versus fecal immunochemical testing in first-degree relatives of patients with non-syndromic colorectal cancer: A multicenter, open-label, parallel-group, randomized trial (ParCoFit study)
Source: PLoS Med. 2023 Oct 24;20(10):e1004298. doi: 10.1371/journal.pmed.1004298 (PMC10597530; doi:10.1371/journal.pmed.1004298)
Supplement: S2 Table — (DOCX) [file pmed.1004298.s006.docx]

**S2 Table. Screening uptake in the FIT^a^ and colonoscopy groups according to whether same/one or different strategies were assigned to the family.**

| **First-degree-relatives** | **Colonoscopy group** | **FIT group** | **Total** | **OR^b^ (95% CI^c^)** | **p value** |
| --- | --- | --- | --- | --- | --- |
| **Same/one strategy assigned in the family** | N= 137 | N = 129 | N = 266 |  |  |
| Screening uptake, n (%) | 58 (42.3) | 56 (43.4) |  | 0.95 (0.58, 1.55) | 0.859 |
| **Different strategies assigned in the family** | N = 294 | N= 310 | N = 604 |  |  |
| Screening uptake, n (%) | 89 (30.3) | 102 (32.3) |  | 0.88 (0.62, 1.24) | 0.487 |

^a^ FIT = fecal immunochemical test

^b^ OR = Odds ratio

^c^ CI = confidence intervals
