# Supplementary material for: Screening uptake of colonoscopy versus fecal immunochemical testing in first-degree relatives of patients with non-syndromic colorectal cancer: A multicenter, open-label, parallel-group, randomized trial (ParCoFit study)
Source: PLoS Med. 2023 Oct 24;20(10):e1004298. doi: 10.1371/journal.pmed.1004298 (PMC10597530; doi:10.1371/journal.pmed.1004298)
Supplement: Attachment — Submitted filename: Review letter.docx [file pmed.1004298.s008.docx]

This paper addresses a crucial concerning the adherence to FIT screening in individuals at high-risk for familial colorectal cancer. This study found that the screening uptake to FIT was not higher than the uptake to colonoscopy, resulting in a lower detection rate of advanced colorectal neoplasia in FIT group than in the colonoscopy group. However, there are concerns regarding a couple of issues in the study.

Major issue:

1. The inclusion of screen-naïve FDR may have introduced selection bias as these individuals have not yet participated in any colorectal cancer screening program. It is possible that these individuals are less likely to participate in screening at all. This low participation rate is evident as only 40% of all individuals agreed to participate in the study. These issues should be discussed in the paper’s discussion section. Additionally, it is worth noting that the participation rate is higher in the FIT group than in the colonoscopy group if calculated based on the number of individuals who agreed to participate (158/187 vs 147/196). This finding is interesting and should be discussed in the paper. Furthermore, a large number of unplanned FIT was found in the colonoscopy group. Were those FITs performed as screening tool, or because of symptoms? If performed as screening tool, that may imply a preference for FIT in at least part of the individuals. This should also be discussed in the paper.

Minor issues:

1. In the introduction, it is mentioned that less than 50% of FDR of patients with colorectal cancer undergo colonoscopy screening. Please clarify this type of screening. Is it 10-yearly colonoscopy?
2. How well is the registration of colonoscopies and FIT outside of this study? Are FITs performed via the screening program included in this study? And why is a FIT performed as a screening tool only included as unplanned FIT in the colonoscopy group? How is a FIT performed via the screening program in the FIT group than included?
3. The paper does not provide a clear indication whether colonoscopies performed in the context of screening are always free of charge in Spain. If they are not, the screening uptake for colonoscopy may be lower than reported in the study. Furthermore, it is unclear whether the colonoscopies performed after a positive FIT result were free of charge. These factors should be taken into account and discussed in the paper.
4. How many individuals in both groups did not attend the initial appointment, but were considered as eligible? Did this number differ between groups?
5. The reason for non-compliance needs to be added to the flowchart, as this is currently unclear. In addition, 1 person is missing from the flowchart in the colonoscopy group. 147 underwent screening in that group, and 48 were non-compliant. That makes a total of 195, but 196 individuals attended the appointment.
6. The tables containing the multivariable logistic regression models require further elaboration. The results of a univariable analysis should be included. Additionally, the statement in the paper that "after adjusting for demographic characteristics, we found that screening uptake was similar in both groups" is not reflected in Table 4. Based on the results in table 4, you can make a statement like: screening strategy, gender, age and participating centers were not significantly associated with screening uptake. Please change the table accordingly to make a statement like: screening uptake was not significantly different between groups, also when adjusting for other variables. In addition, why were only those 3 demographic variables included in the multivariable analysis, and not, e.g. inclusion criteria (one index case < 60 etc.)? I am also curious to see the effect of that variable.
7. In Table 7, the number of individuals who are compliers with the assigned strategy needs to be accompanied by a definition of what constitutes compliance. As this is not clear in the paper, especially as these numbers are not presented in the flowchart. For FIT, is someone a complier after completing 1, 2 or 3 FITs?
8. In Table 7, the number of individuals under post-polypectomy surveillance is much higher in the colonoscopy group. How is that possible? And should those individuals not be excluded from the analyses, as post-polypectomy surveillance is different from screening?
9. Please check the numbers in Table 7, as the numbers in the FIT group do not add up to the total number of compliant/non-compliant.
10. Table 7 needs to clarify when individuals are deemed compliant or non-compliant. Is it only when they did not attend the initial appointment? Individuals in the non-compliant group underwent some type of screening, but it is not clear from the text/table.
11. The discussion states that ‘only 34.9% of participants were randomly assigned to different strategies within the same family’. This is a substantial percentage and may have influence the participation rate. The paper should provide further hypotheses about this factor.
