# Supplementary material for: Screening uptake of colonoscopy versus fecal immunochemical testing in first-degree relatives of patients with non-syndromic colorectal cancer: A multicenter, open-label, parallel-group, randomized trial (ParCoFit study)
Source: PLoS Med. 2023 Oct 24;20(10):e1004298. doi: 10.1371/journal.pmed.1004298 (PMC10597530; doi:10.1371/journal.pmed.1004298)
Supplement: Attachment — Submitted filename: Response to Reviewers.doc [file pmed.1004298.s009.doc]

**RESPONSE TO EDITOR AND REVIEWERS**

**REQUESTS FROM THE EDITOR:**

**GENERAL**

Thank your detailed and considered responses to previous editor and reviewer comments. Please respond to all editor and reviewer comments detailed below in full. Please include line numbers in your revised version, beginning at 1 and in continuous sequence thereafter.

*** The reviewers suggest further analyses, please see below, which have the potential to alter the interpretation of your data and for this reason we have requested a 2nd major revision. ***

**ABSTRACT**

Background:

Suggest ‘we investigated’ instead of ‘we analyzed’

Should the word ‘prevention’ be replaced with ‘detection’ in the final line? Similarly in the author summary (please see below).

Reply: as suggested by the editor we have replaced the word “analyzed” by “investigated” in page 3 (line 7). Also, “detection” was replaced by “detection” in line 9.

Methods and findings:

Please remove the word ‘prospective’ which better describes a cohort study as opposed to a randomized trial .

Reply: we agree with the editor, the word “prospective” has been removed in page 3 (line 10)

Primary outcome – please detail ‘colonoscopy and FIT’ instead of ‘both strategies’

Reply: as suggested by the editor we included ‘colonoscopy and FIT’ in page 3 (line 14)

Please use lower case p to depict the p values.

Reply: lower case was used to depict the p values in the Abstract (page 4, line 28) and throughout the manuscript

Conclusions:

Suggest, ‘In this study, compared to colonoscopy, FIT screening did not improve screening uptake by individuals at high risk of CRC, resulting in less detection of advanced colorectal neoplasia. Further studies are needed to assess how screening uptake could be improved in this high-risk group, including by inclusion in population-based screening programs.

Reply: we reworded the conclusion as suggested by the editor (page 4, lines 32-36)

**AUTHOR SUMMARY**

Why was this study done?

Suggest combining points 1 and 2 to read as follows:

‘The risk of colorectal cancer (CRC) is three to four times higher in first degree relative (FDR) of patients with non-syndromic colorectal cancer. These individuals are considered candidates for colonoscopy-based screening starting at 40 years of age, but this approach is associated with a suboptimal acceptance rate of approximately 50%.’

Reply: points 1 and 2 were combined as suggested by the editor (page 5, lines 48-52)

Please include the abbreviation ‘(CRC)’ before first use later

Point 3 – suggest ‘acceptance of’

Point 4 – should ‘prevention’ be ‘detection’ here?

Reply: clarifications of points 3 and 4 were completed (page 5, lines 62 and 68)

**What did the researchers do and find?**

Point 1 - please split into 2 bullet points at sentence beginning ‘FDR…’

Reply: Point 1 was split into 2 bullet points (page 5, lines 67-71)

Point 2 – suggest removing ‘contrary to expectations’ and making the remaining sentences into 2 points as follows: ‘The rate of screening completion was similar in the group assigned to FIT and the group assigned to colonoscopy screening (36% vs 34% respectively).

‘The detection rate of advanced colorectal neoplasia was significantly lower in subjects receiving annual FIT than in those assigned to receive one-time colonoscopy’.

Reply: we modified point 2 as suggested by the editor (page 5, lines 73-76)

**What do these findings mean?**

In point 1 you state, ‘The findings of this trial indicate that FIT does not have the capacity of increasing colonoscopy screening completion rates in the non-syndromic familial colorectal cancer population.’ But this is inconsistent with the defined hypothesis, ‘that uptake of FIT screening is superior to that of colonoscopy screening in this population’.

Suggest rephrasing for clarity here and to ensure consistency throughout.

Reply: as suggested by Reviewer #1 (see below) we rephrased point 1 for clarification:

- The findings of this trial indicate that FIT does not have the capacity of increasing the acceptance of screening in the non-syndromic familial colorectal cancer population (page 5, lines 84-85)

Point 2 – please remove the word ‘Meanwhile’ and make sentence beginning ‘Future studies are…’ a separate bullet point.

Reply: point 2 has been modified as suggested by the editor

**INTRODUCTION**

Sentence beginning, ‘Recently, a large prospective study…’ suggest making a new paragraph

Reply: as suggested by the editor we have rephrased this paragraph (please see page 7, lines 106-110)

Page 6 final paragraph – suggest avoiding repeated use of the word ‘recently’

Reply: the word recently was deleted (please see page 8, line 129 in the revised marked-up version)

Final sentence - ‘…test the hypothesis that uptake of FIT screening followed by a work-up colonoscopy in the case of a positive test is superior to colonoscopy screening with an equivalent effect on colorectal cancer detection in the population with high familial risk’ this is again different to the abstract and the author summary. We think that you are trying to say ‘uptake of FIT screening followed by a work-up colonoscopy…is superior to uptake of one-time colonoscopy screening…’ as we understand things. Suggest revising throughout all sections to ensure that the hypothesis is clearly and consistently defined for the reader.

Reply: we apologize for this confusion. We ensured that the hypothesis has the same wording in all sections (please see page 3 - line 8, page 5 - line 67 and page 8 - line 138)

**METHODS and RESULTS**

Please define the acronym ‘CONSORT’ for the reader, as in your previous version, my apologies for not making this clear previously.

Please also see reviewer comments detailed below regarding additional analyses required, which we agree with.

Reply: we defined the acronym CONSORT in page 9, line 163.

We performed the additional analysis requested by Reviewer #5 (please, see below)

**FIGURES**

Figure 1 – please ensure all abbreviations are defined for the reader in an appropriate footnote/caption.

Reply: Fig 1 was revised, and appropriate footnote/caption were added, complying with Plos Medicine standards.

**TABLES**

The terms ‘male and female gender’ are used incorrectly (table 1, for example). ‘Gender’ should be replaced with ‘Sex’. Alternatively, simply detailing ‘Male n (%)’ and ‘Female n (%)’ would suffice.

Please check and revise throughout all tables including those in the supporting files as appropriate.

Reply: we apologize for the mistake. We have corrected the error in all Tables

**DISCUSSION**

Line 1 – suggest removing the word ‘pragmatic’.

Reply: we have removed the word “pragmatic” in page 41, line 524.

**REFERENCES**

For in-text reference callouts please remove the space between different citations to read as follows, [1,3,6] (as opposed to [1, 3, 6]). Please check and amend throughout all sub-sections of the manuscript and supporting information.

Reply: we have removed the space between different citations following your instructions.

**SUPPORTING INFORMATION**

CONSORT checklist – please remove the numbers 1 and 2 from the first 2 lines (as these refer to page numbers). ‘Title’ and ‘Abstract’ will suffice.

Reply: numbers 1 and 2 were removed from the first 2 lines

**STATISTICAL ANALYSIS PLAN** – please ensure that the reference formatting follows our guidance, as for the main manuscript, including in-text reference callouts.

Please note that we cannot publish copyright symbols such as ©, ®, or ™ (i.e. OC-Sensor ® kit) please revise throughout where relevant including the main manuscript.

Reply: we revised throughout the manuscript and deleted the copyright symbols.

**REVIEWER #1**

I still have some comments to your revised manuscript:

**Abstract and author summary:**

-You use the term advanced colorectal neoplasia without defining it. It is defined later in the methods but should be defined here as well.

Reply: as requested by the Reviewer, the term advanced colorectal neoplasia was defined in the abstract and in the introduction (page 3 lines 15-17, page 7 lines 107-108).

**Author summary**

-"What do these findings meet", I see that the statement "What do these findings mean" is changed, but I still find it not clear. "The findings of this trial indicate that FIT does not have the capacity of increasing the acceptance of colonoscopy screening in the non-syndromic familial colorectal cancer population. " In my opinion this should be rephrased: "The findings of this trial indicate that FIT does not have the capacity of increasing the acceptance of screening in the non-syndromic familial colorectal cancer population."

Reply: we thank the reviewer for this clarification. This sentence was rephrased accordingly (please see page 5, lines 86-87).

**Methods/Study procedures:**

- Grammatical error: "Colon cleansing were performed as previously described [19]." must be changed to "was" performed.

Reply; this error was corrected in Page 10, line 195.

-"They were notified to deliver it to the laboratory within 14 days." I think it should be clarified that what you mean is that they were notified to deliver it within 14 days after taking the sample and not within 14 days after receiving the kit.

Reply: we thank the reviewer for this clarification. We have rephrased this on page 11, lines 205-206.

-Grammatical error: "The individuals who did not deliver the test on time were contacted by telephone for offer them a new test" should be "to" offer

Reply; this error was corrected in Page 11, line 211-212.

-"We considered severe post-polypectomy bleeding if prevented the conclusion of the procedure o transfusion was required." Is what you mean: that the completion of the procedure was prevented or blood transfusion was required? What about hospitalization or repeated endoscopy? That was not a criteria?

Reply: it is true that most patients with severe postpolypectomy bleeding requires hospitalization. We added hospitalization to this phrase (page 12, line 224). All cases in which colonoscopy was not completed due to post-polypectomy hemorrhage were subsequently rescheduled.

Results:

- Table 5: please check the WHO definition for sessile serrated lesions. Now you changed the changed the term but it is not correct. You wrote in your answer that there was no dysplasia and therefor there were no advanced serrated lesions. But you write in the table sessile serrated adenoma. This term does not exist. All adenomas have dysplasia. I think what you mean is non-advanced sessile serrated lesions. Is it correct that no traditional serrated adenomas were detected (all TSA have dysplasia).

Reply: we thank the Reviewer for detecting this error. We have corrected it accordingly in Table 5.

General:

-there are some spelling errors, double words, missing commas throughout the manuscript.

Reply: we corrected spelling errors, double words and missing commas throughout the manuscript.

**REVIEWER #2:**

Thanks to the authors for addressing my previous comments. I just wanted to highlight that describing the randomisation as 'simple randomisation' and 'stratified by centre' is slightly contradictory. I assume that stratified block randomisation was used? Simple randomisation would just be randomly allocating each individual with 50% probability.

Reply: we thank the reviewer for detecting this error. We really performed stratified block randomization. We stratified the randomization by each group (hospital) site. We have corrected it accordingly in the Abstract and Methods. Please see page 3, line 20 and page 10, line 176.

**REVIEWER #5:**

The authors have improved the manuscript and incorporated most of my comments. However, I do have one follow-up comment that needs to be addressed before publication.

The authors now state in the rebuttal as well as in the Discussion: "Consequently, some individuals had a different screening strategy assigned in the same family, which could have led to refusal to participate in some cases. In fact, the assignment of different strategies to members of the same family was an independent factor for low participation in the logistic regression analysis. However, the rate of subjects who were assigned to different strategies in the same family was similar in the FIT group (51.3%), and in the colonoscopy group (48.7%), suggesting that this condition affected both groups equally."

This statement cannot be made based on these results only. You can only state that the rate of subjects who were assigned to difference strategies in the same family was similar in both groups. However, it may be possible that, for example, those who were assigned to FIT, were less likely to participate with FIT, because family members were assigned to colonoscopy (and thereby being less comfortable with less invasive surveillance).

In order to make this statement, the effect of different strategies assigned should be analysed separately for the FIT and the colonoscopy group: thus, what is the screening uptake in the FIT group stratified for those assigned same and different strategy, and the uptake in the colonoscopy group stratified for those assigned same and different strategy. If those results show similar results in participation rates, I agree with the statement. However, if those results show differences in participation rates, it should be clearly stated that this is likely to have affected the results, and make definitive conclusions about the difference in participation between annual FIT and colonoscopy complicated.

Reply: we thank the reviewer for raising this relevant question. As per the reviewer's request, we analyzed screening uptake separately for the FIT and colonoscopy groups. We stratified the analysis by considering whether the same strategy or different strategies were assigned to the family (please, see below).

In the FIT group, 56/129 (43,4%) FDR belonging to families that were assigned to the same strategy underwent FIT screening, whereas 102/319 (32,9%) FDR assigned to different strategies underwent FIT screening.

| **FIT GROUP: Screening uptake stratified by Same vs Different strategy assignment in the family, according to intention-to-screen analysis - Crosstabulation** | | | | | | |
| --- | --- | --- | --- | --- | --- | --- |
|  | | | **Screening Uptake** | | Total |  |
|  |  |  | NO | YES |  |  |
| Same/One vs different strategies in the family | **Same or One strategy** | Count | 73 | 56 | 129 |  |
|  |  | % within Same vs different strategy in the family | 56,6% | 43,4% | 100,0% |  |
|  | **Different strategies** | Count | 208 | 102 | 310 |  |
|  |  | % within Same vs different strategy in the family | 67,1% | 32,9% | 100,0% |  |
| Total | | Count | 281 | 158 | 439 |  |
|  |  | % within Same vs different strategy in the family | 64,0% | 36,0% | 100,0% |  |

In the colonoscopy group, 58/137 (42.3%) FDR assigned to the same strategy underwent colonoscopy screening, whereas 89/294 (30.3%) of those assigned to different strategies received colonoscopy screening.

| **COLONOSCOPY GROUP: Screening uptake stratified by Same vs Different strategies assignment in the family, according to intention-to-screen analysis - Crosstabulation** | | | | | | | |  |
| --- | --- | --- | --- | --- | --- | --- | --- | --- |
|  | | | **Screening Uptake** | | | | Total |  |
|  |  |  | NO | YES | | |  |  |
| Same/One vs different strategies in the family | **Same or One strategy** | Count | 79 | | 58 | 137 | | |
|  |  | % within Same vs different strategy in the family | 57,7% | | 42,3% | 100,0% | | |
|  | **Different**  **strategies** | Count | 205 | | 89 | 294 | | |
|  |  | % within Same vs different strategy in the family | 69,7% | | 30,3% | 100,0% | | |
| Total | | Count | 284 | | 147 | 431 | | |
|  |  | % within Same vs different strategy in the family | 65,9% | | 34,1% | 100,0% | | |

Screening uptake in the FIT group was similar to the screening uptake in the colonoscopy group in FDR that were assigned the same strategy per family (OR 0.95 (0.58, 1.55, p=0.859). Similarly, screening uptake did not differ between arms, in the subgroup of FDR that were assigned different strategies to the family (OR 0.88, 95% CI [0.62, 1.24], p=0.487). We included these results in a new S2_Table, that replaced the previous one.

**S2_table. Screening uptake in the FIT and colonoscopy groups according to whether same/one or different strategies were assigned to the family.**

| **First-degree-relatives** | **Colonoscopy group** | **FIT group** | **Total** | **OR (95% CI)** | **p value** |
| --- | --- | --- | --- | --- | --- |
| **Same/one strategy assigned to the family** | N= 137 | N = 129 | N = 266 |  |  |
| Screening uptake, n (%) | 58 (42.3) | 56 (43.4) |  | 0.95 (0.58, 1.55) | 0.859 |
| **Different strategies assigned to the family** | N = 294 | N= 310 | N = 604 |  |  |
| Screening uptake, n (%) | 89 (30.3) | 102 (32.3) |  | 0.88 (0.62, 1.24) | 0.487 |

We have added a summary of this analysis in the Results and Discussion sections of the manuscript (please, see page 26, lines 393-397, and page 42 , lines 561-563, respectively).
