# Supplementary material for: Screening uptake of colonoscopy versus fecal immunochemical testing in first-degree relatives of patients with non-syndromic colorectal cancer: A multicenter, open-label, parallel-group, randomized trial (ParCoFit study)
Source: PLoS Med. 2023 Oct 24;20(10):e1004298. doi: 10.1371/journal.pmed.1004298 (PMC10597530; doi:10.1371/journal.pmed.1004298)
Supplement: Attachment — Submitted filename: Requests from Editors 07-09-23.docx [file pmed.1004298.s010.docx]

**Requests from Editors:**

GENERAL
Thank you for your considered and detailed responses to previous editor and reviewer comments. Please see below for further comments which we require you address prior to publication.

COMPETING INTERESTS
Please ensure that competing interests have been declared as per the PLOS policy, which can be seen here:
<https://journals.plos.org/plosmedicine/s/competing-interests>
For authors with ties to industry, please indicate whether any of the interests has a financial stake in the results of the current study.

Reply: competing interests have been added as per PLOS policy

ABSTRACT
In the last sentence of the Abstract Methods and Findings section, please describe the main limitation(s) of the study's methodology.

Reply: the sentence “The main limitation of this study was the impossibility of collecting information on eligible individuals who declined to participate” , has been added in the Abstract (please see lines 24-25)

AUTHOR SUMMARY
Line 74 – suggest ‘is that it was…’ also suggest expanding (very briefly) to detail that this impedes understanding of the very low screening uptake across both study arms. Perhaps, ‘The main limitation of this study is that it was not possible to collect information on eligible individuals who declined to participate thus impeding our understanding behind low screening uptake in this population.’ Or similar.

Reply: As suggested by the editor we clarified this sentence adding ¨…..thus impeding our understanding behind low screening uptake in this population.” (please, see lines 77-78).

INTRODUCTION
Line 99 – please remove the underscore preceding the opening bracket which appears to be a typographical error.

Reply: the error has been corrected (please, see line 100)

METHODS and RESULTS
Line 175 – please replace the term, ‘OC-Sensor kit’ with ‘quantitative FIT (Fecal Immunochemical Test)’ as the former is a trademark label. Please check and amend throughout. You could elaborate to describe the test ‘designed to detect occult human haemoglobin in human stool’ or similar perhaps.

Reply: OC-Sensor was substituted by “automated quantitative FIT (Fecal Immunochemical Test)” (now lines 176-177)

Line 222 – suggest ‘were’ instead of ‘was’.
Reply: the error was corrected (please see line 224)

TABLES
Table 1 – is there a way to reduce the space between n numbers and percentages in columns 2 and 3? This would improve accessibility to the reader.

Table 2 – line numbers overwrite the data in the final column, bottom 4 rows.

Table 5 – final row, penultimate column, please replace the hyphen with a comma.

Reply: errors in tables 1, 2 and 5 have been corrected

SUPPORTING INFORMATION
The supporting information name and number are required in a caption, and we highly recommend including a one-line title as well.

Reply: we have added a caption “ParCoFit study database” for the supporting information, at the end of the manuscript

You may also include a legend in your caption, but it is not required.
In the published article, supporting information files are accessed only through a hyperlink attached to the captions. For this reason, you must list captions at the end of your manuscript file. You may include a caption within the supporting information file itself, as long as that caption is also provided in the manuscript file. Do not submit a separate caption file.
Please revise the ‘Supplementary Information Files’ list on page 46 of the manuscript PDF to detail instead only the relevant titles/captions for the figures/tables.

STUDY PROTOCOL
* Page 4: as above, please replace, ‘(OC-Sensor™)’ with ‘quantitative FIT (Fecal Immunochemical Test), designed to detect occult human haemoglobin in human stool’. Please check and amend throughout as the former is trademark label. Please check and amend throughout.

Reply: OC-Sensor^TM^ was substituted by “automated quantitative Fecal Immunochemical Test” in page 4

* Please replace hyphens with commas when reporting 95% CIs as the former can be confused with reporting of negative values. I note that on occasion the word 'to' is also used. Please check and amend throughout for consistency and clarity.

Reply: changes were performed as requested.

* Please ensure that for in-text reference callouts, citations are placed in square brackets as for the main manuscript. Please check and amend throughout all supporting files.

Reply: changes were performed as requested. All citations are placed in square brackets.

* Page 5, para 3: please replace the full stop with a comma when reporting citations, ‘[10,11]’ (as opposed to ‘(10.11)’).

Reply: The full stop was replaced with a comma.

* References – please ensure that the reference formatting follows our guidance as for the main manuscript. Please see here for further details <https://journals.plos.org/plosmedicine/s/submission-guidelines#loc-references>. Please list up to but no more than 6 author names followed by et al in the event that more than 6 authors contribute to a study.

Reply: the reference formatting was modified according to Plos Medicine guidance

SOCIAL MEDIA
To help us extend the reach of your research, please detail any Twitter handles you wish to be included when we tweet this paper (including your own, your coauthors’, your institution, funder, or lab) in the manuscript submission form when you re-submit the manuscript.

Reply: Twitter address: #EQUINTEROC


Comments from Reviewers:

Reviewer #1: Thanks to the authors for addressing my previous comments.


Reviewer #5: Thanks to the authors for performing the requested analysis. These results indeed clearly show that, although the overall screening uptake was signficantly lower in those assigned different strategies (which the authors already state based on the logistic regression analysis), but that the screening uptake of colonoscopy did not significantly differ from the screening uptake of FIT in each of the two categories.


Any attachments provided with reviews can be seen via the following link:
